# Tumor suppressor p53 restrains cancer cell dissemination by modulating mitochondrial dynamics

**DOI:** 10.1038/s41389-022-00401-x

**Published:** 2022-05-19

**Authors:** Trinh T. T. Phan, Yu-Chun Lin, Yu-Ting Chou, Chien-Wei Wu, Lih-Yuan Lin

**Affiliations:** 1 Institute of Molecular and Cellular Biology, College of Life Science, National Tsing Hua University, Hsinchu, 300044 Taiwan ROC; 2 Institute of Molecular Medicine, College of Life Science, National Tsing Hua University, Hsinchu, 300044 Taiwan ROC; 3 Institute of Biotechnology, College of Life Science, National Tsing Hua University, Hsinchu, 300044 Taiwan ROC

**Keywords:** Cell migration, Cell signalling, Metastasis

## Abstract

Tumor suppressor p53 plays a central role in preventing tumorigenesis. Here, we unravel how p53 modulates mitochondrial dynamics to restrain the metastatic properties of cancer cells. p53 inhibits the mammalian target of rapamycin complex 1 (mTORC1) signaling to attenuate the protein level of mitochondrial fission process 1 (MTFP1), which fosters the pro-fission dynamin-related protein 1 (Drp1) phosphorylation. This regulatory mechanism allows p53 to restrict cell migration and invasion governed by Drp1-mediated mitochondrial fission. Downregulating p53 expression or elevating the molecular signature of mitochondrial fission correlates with aggressive tumor phenotypes and poor prognosis in cancer patients. Upon p53 loss, exaggerated mitochondrial fragmentation stimulates the activation of the extracellular signal-regulated kinase 1/2 (ERK1/2) signaling resulting in epithelial-to-mesenchymal transition (EMT)-like changes in cell morphology, accompanied by accelerated matrix metalloproteinase 9 (MMP9) expression and invasive cell migration. Notably, blocking the activation of mTORC1/MTFP1/Drp1/ERK1/2 axis completely abolishes the p53 deficiency-driven cellular morphological switch, MMP9 expression, and cancer cell dissemination. Our findings unveil a hitherto unrecognized mitochondria-dependent molecular mechanism underlying the metastatic phenotypes of p53-compromised cancers.

## Introduction

Metastasis remains the biggest challenge in cancer treatment and the major cause of cancer-related deaths. Recently, mitochondrial dynamics has been implicated in controlling the metastatic dissemination of cancer cells [1–5]. Enforcing mitochondrial fission or inhibiting mitochondrial fusion supports cell migration, invasion, and metastasis in hepatocellular carcinoma, glioma, pancreatic, and breast cancers. Paradoxically, increased mitochondrial fission attenuates metastasis in triple-negative breast cancer [6]. Thus, whether mitochondrial fission or fusion advances the metastatic potential of cancer cells may be context-dependent and requires further investigation.

It has been established that the mammalian target of rapamycin complex 1 (mTORC1) is an important regulator of mitochondrial dynamics [7]. mTORC1 phosphorylates 4E-BPs (the translation initiation factor 4E (eIF4E)-binding proteins) and prevents it from binding eIF4E [8–10]. The eIF4E can then initiate the translation of the mitochondrial fission process 1 (MTFP1) [7]. MTFP1 is a transmembrane protein located in the mitochondrial inner membrane and facilitates mitochondrial fission [7, 11, 12]. Studies have suggested mTORC1 activation contributes to elevated cancer migration, invasion, and metastasis [13–15], while mTOR inhibition results in mitochondrial elongation and branching [7], and the morphology can be completely reversed by overexpressing MTFP1 [7]. However, the links among mTOR, metastasis, and mitochondrial dynamics have not been examined.

The tumor suppressor p53, encoded by the tumor protein p53 (*TP53*) gene, is a master regulator of multiple cell fate-determining genes and prevents the oncogenic activation of the mTOR signaling pathway [16–23]. Accumulating data suggests an unconventional role of p53 in controlling cancer cell invasiveness [24]. p53 also impacts mitochondrial integrity in response to various stresses by either regulating proteins involved in mitochondrial quality control or maintaining the mitochondrial genomic integrity [25–27]. Nonetheless, how p53 modulates the morphological dynamics of mitochondria remains poorly understood. Moreover, whether mitochondrial dynamics is involved in p53-dependent regulation of cell motility and invasion has not been addressed.

In this study, we delineate a p53-regulated circuitry that restrains the metastatic dissemination of cancer cells and contributes to cancer phenotypes and patient prognosis. We show that p53 alleviates the dynamin-related protein 1 (Drp1)-driven mitochondrial fission by inhibiting the mTORC1-mediated MTFP1 protein expression. p53 deficiency-exaggerated mitochondrial fragmentation activates the extracellular signal-regulated kinase 1/2 (ERK1/2) signaling leading to remarkable changes in cell morphology and robust increases in the matrix metalloproteinase 9 (MMP9) expression and invasive cell migration. Hence, mitochondrial fission represents a driving force for signal transduction that directs cancer cell migration and invasion when wild-type (WT) p53 functions are impaired.

## Results

### Downregulation of WT p53 expression is associated with aggressive tumor phenotypes and poor prognosis

**Fig. 1:**
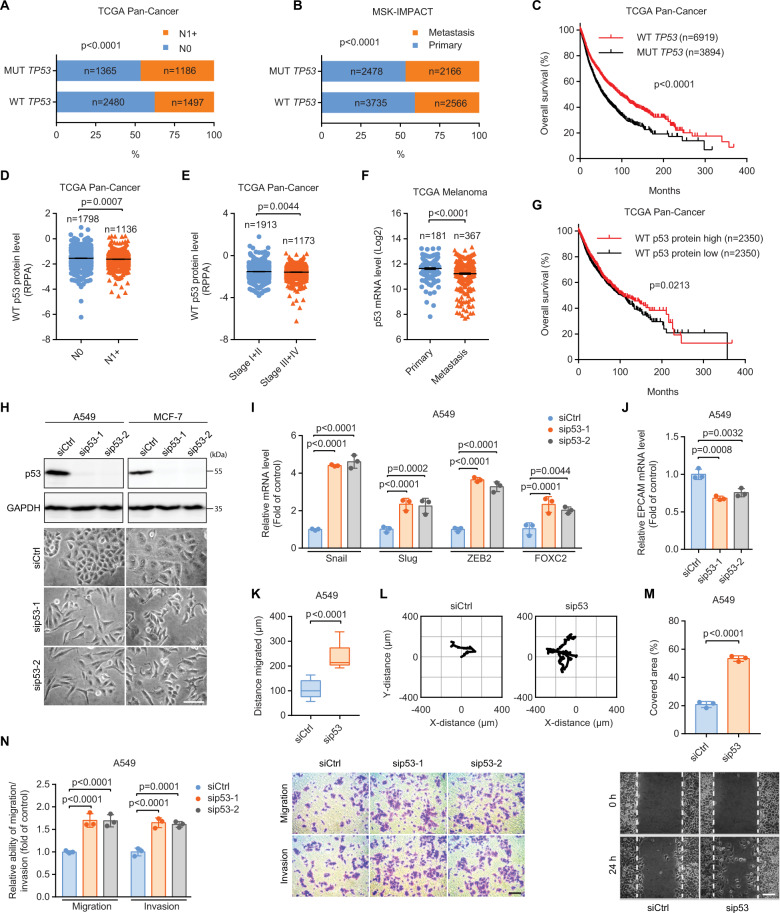
Downregulation of WT p53 expression is associated with aggressive tumor phenotypes and poor prognosis. Contingency analysis of the associations between the presence of *TP53* mutations and the probabilities of metastases to (**A**) lymph nodes and (**B**) distant organs. N0, lymph node-negative; N1+, lymph-node-positive. Data were derived from (**A**) The Cancer Genome Atlas (TCGA) Pan-Cancer and (**B**) the Memorial Sloan-Kettering Integrated Mutation Profiling of Actionable Cancer Targets (MSK-IMPACT) cohorts. The *p* values represent the significance of the observed mutual exclusivity between the WT *TP53* and MUT *TP53* groups (**A** and **B**). **C** Kaplan–Meier analysis of overall survival in cancer patients having WT and MUT *TP53*. p53 protein expression of patients having WT *TP53* (**D**) with N0 and N1+ and (**E**) with stage I + II and III + IV tumors. RPPA, reverse-phase protein array. **F** p53 mRNA levels in primary and distant metastatic melanoma. **G** Kaplan–Meier analysis of overall survival in *TP53* WT cancer patients with low and high p53 protein levels. Data were extracted from TCGA (**C**–**G**). **H** Phase-contrast imaging of control (siCtrl) and p53-silenced (sip53-1 and sip53-2) A549 and MCF-7 cells. Scale bar: 100 µm. qRT-PCR analysis of the mRNA levels of (**I**) EMT inducers Snail (Snai1), Slug (Snai2), ZEB2 (zinc finger E-box binding homeobox 2), and FOXC2 (forkhead box protein C2) and (**J**) the epithelial cell adhesion molecule EPCAM in siCtrl, sip53-1, and sip53-2 A549 cells. Migration distance (**K**) and representative trajectories (**L**) of siCtrl (*n* = 28) and sip53 (*n* = 14) A549 cells. **M** Quantification (top) and representative images (bottom) of the area in a wound-healing assay covered by A549 cells transfected with siCtrl or sip53. Scale bar: 100 µm. **N** Transwell assays for siCtrl, sip53-1, and sip53-2 A549 cells. Scale bar: 100 µm. Error bars represent mean ± SEM (**D**–**F**) or SD (**I**, **J**, **M**, and **N**). Data were analyzed by Fisher’s exact test (**A** and **B**), log-rank test (**C** and **G**), two-tailed unpaired Student’s *t* test (**D**–**F**, **K**, and **M**), or one-way ANOVA with Tukey’s multiple comparisons test (**I**, **J**, and **N**).

Given that *TP53* is among the most frequently altered genes in metastatic cancers [28], we investigated the associations between the presence of *TP53* mutations and cancer metastases using publicly available databases (Fig. 1A, B). Results revealed that cancer patients harboring mutant (MUT) *TP53* had a higher risk of developing metastases to lymph nodes (Fig. 1A, lymph node-negative (N0) vs lymph node-positive (N1+)) and distant organs (Fig. 1B) as compared to those having WT *TP53*. In addition, median overall survival was over 1.7-fold longer in patients with WT *TP53* than in those with MUT *TP53* (Fig. 1C).

Most *TP53* mutations in human cancers are missense mutations [29–31]. Missense mutations in the *TP53* gene not only abrogate the normal tumor-suppressive functions of WT p53 but also exert novel oncogenic gain-of-function activities that exacerbate cancer development and metastasis [30, 32]. A closer analysis of the associations between different *TP53* subtypes and lymph node metastases showed that the group of *TP53* unaltered cancer patients displayed a much smaller fraction of N1+ tumors (~36%) than either the *TP53* missense mutation (~48%) or gene deletion group (~44%) (Fig. S1). Notably, the proportion of N1+ tumors was prominently higher in the *TP53* missense mutation group than in the *TP53* deletion group (Fig. S1). Results suggest that the effect magnitudes of different *TP53* alterations on cancer metastases are not equal. Cancer patients harboring *TP53* missense mutations have a greater likelihood of developing metastases than their *TP53* gene deletion counterparts, potentially via gain-of-function activities exerted by MUT p53 proteins.

As emerging evidence demonstrates the important role of WT p53 in suppressing cancer metastasis [24], we reasoned that the expression level of WT p53 protein might also be a contributing factor in determining disease aggressiveness and patient outcomes. To this end, we analyzed p53 protein levels in primary tumors from N0 and N1+ patients harboring WT *TP53* (Fig. 1D). Results indicated that N1+ tumors had decreased levels of WT p53 protein as compared to those in N0 tumors. Of tumors having WT *TP53* gene, p53 protein levels were also reduced in advanced-stage (III + IV) tumors when compared to those in the earlier-stage (I + II) tumors (Fig. 1E). In line with these observations, p53 mRNA levels were significantly decreased in distant metastatic melanoma compared to those in primary melanoma (Fig. 1F). Reduced p53 protein levels were correlated with impaired overall survival in cancer patients harboring WT *TP53* (Fig. 1G). These data suggest that impaired expression of WT p53 is implicated in exaggerated malignant phenotypes and poor prognosis of cancer patients.

### p53 silencing accelerates cancer cell migration and invasion

To validate the contribution of WT p53 in suppressing cancer dissemination, we silenced p53 in human non‑small cell lung cancer (NSCLC) A549 and human breast cancer MCF-7 cells with small interference (si)RNAs. Both cell types express WT p53. p53 depletion induced significant morphological changes in both A549 and MCF-7 cells (Fig. 1H). p53-depleted cells exhibited a decrease in cell-cell adhesions, an elongated cell body, and a spindle-shaped morphology, which were much different from the high cell-cell adhesion and epithelial-like morphology in p53 WT controls. p53 depletion elevates the mRNA levels of epithelial-to-mesenchymal transition (EMT)-promoting factors (Fig. 1I) but repressed the epithelial cell adhesion molecule EPCAM (Fig. 1J) in A549 cells, suggesting that loss of p53 triggers EMT, a key event that drives cancer metastasis. Moreover, p53 silencing stimulated A549 cell motility (Fig. 1K–N) and invasion (Fig. 1N). Taken together, these results indicate that loss of WT p53 induced a more aggressive cancer cell phenotype and heightened cell motility and invasion.

### p53 silencing amplifies mitochondrial fission has diagnostic and clinical implications

**Fig. 2:**
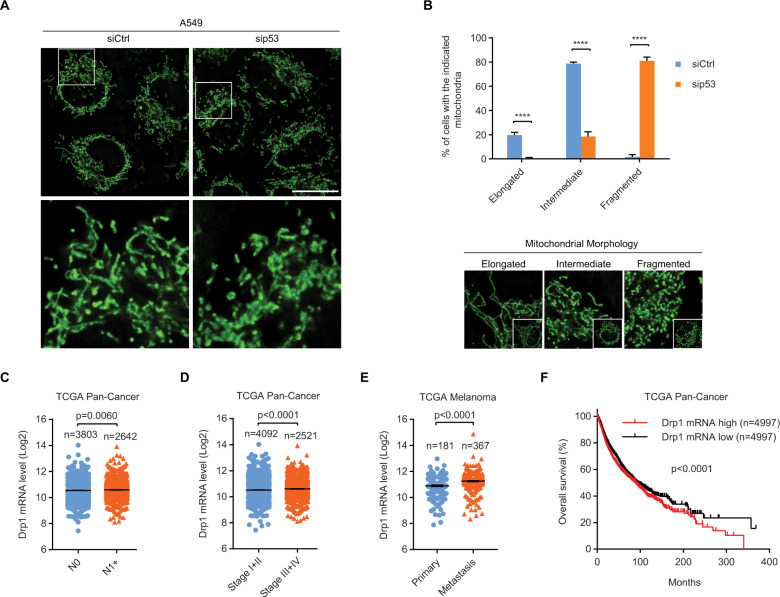
p53 silencing amplifies mitochondrial fission has diagnostic and clinical implications. Representative images (**A**) and quantification (**B**) of mitochondrial morphology in siCtrl (*n* = 222) and sip53 (*n* = 230) A549 cells. Boxed regions in (**A**) are shown enlarged in the bottom panels. Scale bar: 20 µm. **B** Representative images for each mitochondrial morphology type are shown in the bottom panels. Drp1 mRNA expression in (**C**) N0 and N1 + or (**D**) stage I + II and III + IV tumors. **E** Drp1 mRNA levels in primary and distant metastatic melanoma. **F** Kaplan–Meier analysis of overall survival in cancer patients with low and high Drp1 mRNA levels. Data were extracted from TCGA (**C**–**F**). Error bars represent mean ± SD (**B**) or SEM (**C**–**E**). Data were analyzed by one-way ANOVA with Tukey’s multiple comparisons test (**B**), two-tailed unpaired Student’s *t* test (**C**–**E**), or log-rank test (**F**). ****p < 0.0001.

Building on previous findings that metastasizing cancer cells need to alter their mitochondrial morphology to facilitate their motility and invasiveness [1–3], we assessed the morphological dynamics of mitochondria upon WT p53 loss. Using live-cell fluorescence imaging, we observed over 78% of cells harboring WT p53 had intermediate mitochondria and 19% of those cells had elongated mitochondria. Meanwhile, only less than 2% of those cells carried fragmented mitochondria. Upon p53 silencing, the percentage of cells with fragmented mitochondria was enhanced to more than 80% while less than 20% and 1% of p53-depleted cells showed intermediate and elongated mitochondria, respectively (Fig. 2A, B). These results provide strong evidence that mitochondrial dynamics is modulated by p53.

As p53 silencing amplified mitochondrial fission, we investigated the clinical significance of Drp1, a major pro-fission protein. Drp1 mRNA levels were elevated in N1+ (Fig. 2C) and advanced-stage (III + IV) (Fig. 2D) tumors compared to those in N0 and earlier-stage (I + II) tumors, respectively. Consistent with this finding, Drp1 mRNA levels were higher in distant metastatic melanoma than in primary melanoma (Fig. 2E). Furthermore, high Drp1 levels significantly reduced overall survival in cancer patients (Fig. 2F). These data corroborate a strong association of mitochondrial morphology with the degree of tumor malignancy and clinical outcomes in cancers.

### p53 elevation promotes mitochondrial elongation accompanied by attenuated invasive cell migration

**Fig. 3:**
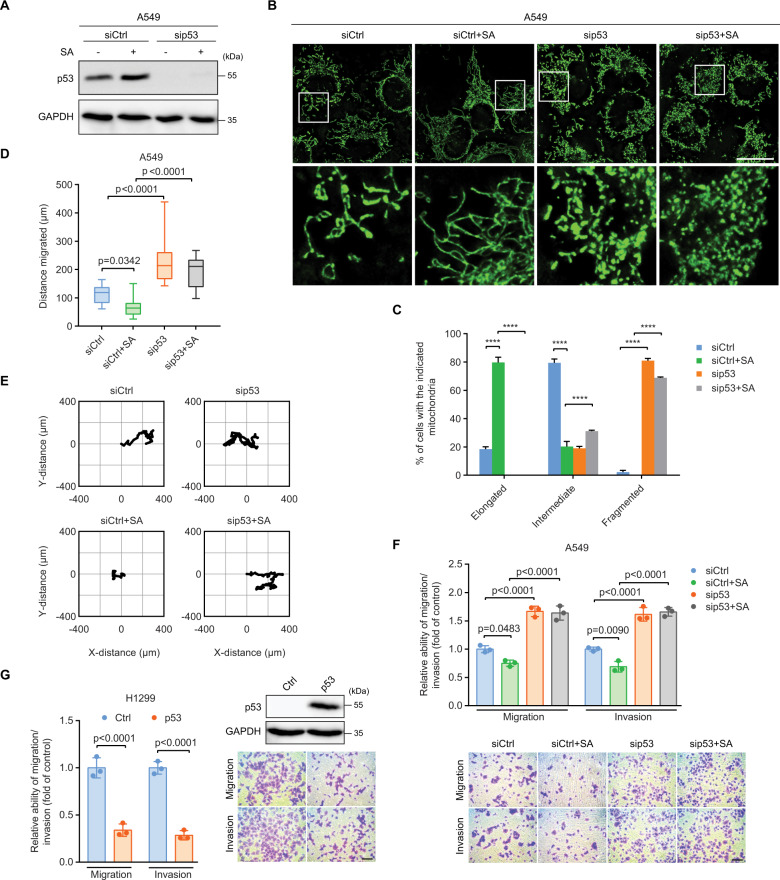
p53 elevation promotes mitochondrial elongation accompanied by attenuated invasive cell migration. **A** Immunoblot of p53 in siCtrl and sip53 A549 cells with and without 20 µM SA treatment for 24 h. GAPDH was used as a loading control. Representative images (**B**) and quantification (**C**) of mitochondrial morphology in siCtrl (*n* = 223), siCtrl+SA (*n* = 211), sip53 (*n* = 205), and sip53+SA (*n* = 218) A549 cells. Boxed regions in (**B**) are shown enlarged in the bottom panels. Scale bar: 20 µm. Migration distance (**D**) and representative trajectories (**E**) of siCtrl (*n* = 29), siCtrl+SA (*n* = 17), sip53 (*n* = 13), and sip53+SA (*n* = 15) A549 cells. Transwell assays for (**F**) siCtrl, siCtrl+SA, sip53, and sip53+SA A549 cells or (**G**) control (Ctrl) and p53-overexpressing (p53) H1299 cells. Scale bar: 100 µm. Error bars represent mean ± SD. Data were analyzed by one-way ANOVA with Tukey’s multiple comparisons test. ****p < 0.0001.

Sodium arsenite (SA) is a genotoxic agent that induces DNA damage in human cells [33, 34] and elevates endogenous p53 expression (Fig. S2A). Although 10 µM SA was sufficient to induce p53 expression in A549 cells (Fig. S2A), treating cells with 10, 20, or 40 µM SA did not affect cell viability and proliferation (Fig. S2B, C). Furthermore, A549 cells treated with increased concentration (20 or 40 µM) of SA had higher accumulations of p53 protein. Thus, a 24-h treatment with a non-cytotoxic concentration of 20 µM SA was chosen to amplify the endogenous p53 expression in A549 cells. The expression of the endogenous p53 with and without SA induction can be effectively silenced with siRNA (Fig. 3A). Critically, these treatments did not change the survival of p53-depleted cells (Fig. S2D).

SA-elevated endogenous p53 expression promoted an over fourfold increase in mitochondrial branching and elongation (Fig. 3B, C). In SA-treated A549 cells, almost 80% of cells showed elongated mitochondria as compared to 20% in control cells. p53 depletion completely reversed the effects of SA on mitochondrial elongation, accompanied by enhanced mitochondrial fragmentation. Over 70% of SA-treated and p53-silenced cells exhibited fragmented mitochondria that were absent in SA-treated only p53 WT cells. Intriguingly, mitochondrial membrane potential (ΔΨm) and levels of mitochondrial reactive oxygen species (ROS) were unaltered by increasing or decreasing the expression of p53 (Fig. S2E, F). These results underscore the central role of p53 in the control of mitochondrial dynamics without affecting mitochondrial integrity.

Notably, SA-treated cells exhibited an over 1.5-fold decrease in migratory capacity as compared to that of control cells (Fig. 3D, E). Knockdown of p53 fully abolished the inhibitory effects of SA on cell migration. p53 silencing accelerated the migratory ability of SA-treated cells to a level similar to that of untreated and p53-depleted cells. Transwell assays also showed that SA lowered cell migration and invasion but these effects were abolished by p53 silencing (Fig. 3F). These results further support the suppressive role of p53 in the metastatic properties of cancer cells.

To further substantiate the role of p53 in regulating cell migration and invasion, we overexpressed exogenous WT p53 in p53-null NSCLC H1299 cells (Fig. 3G). Expression of WT p53 caused an approximately 2.5-fold decrease in the migratory capacity of the cells as compared to that of the controls (Fig. S2G). Transwell assays also showed that expression of WT p53 triggered 3- and 3.5-fold decreases in the migratory and invasive capacity of the cells, respectively, compared to that of the p53-null controls (Fig. 3G). These results, together with the observation that p53 depletion exaggerated mitochondrial fragmentation and invasive cell migration, pinpoint p53 as a potent regulator of mitochondrial dynamics and cell motility.

### p53 alleviates Drp1-mediated mitochondrial fission and thereby restrains cell migration and invasion

**Fig. 4:**
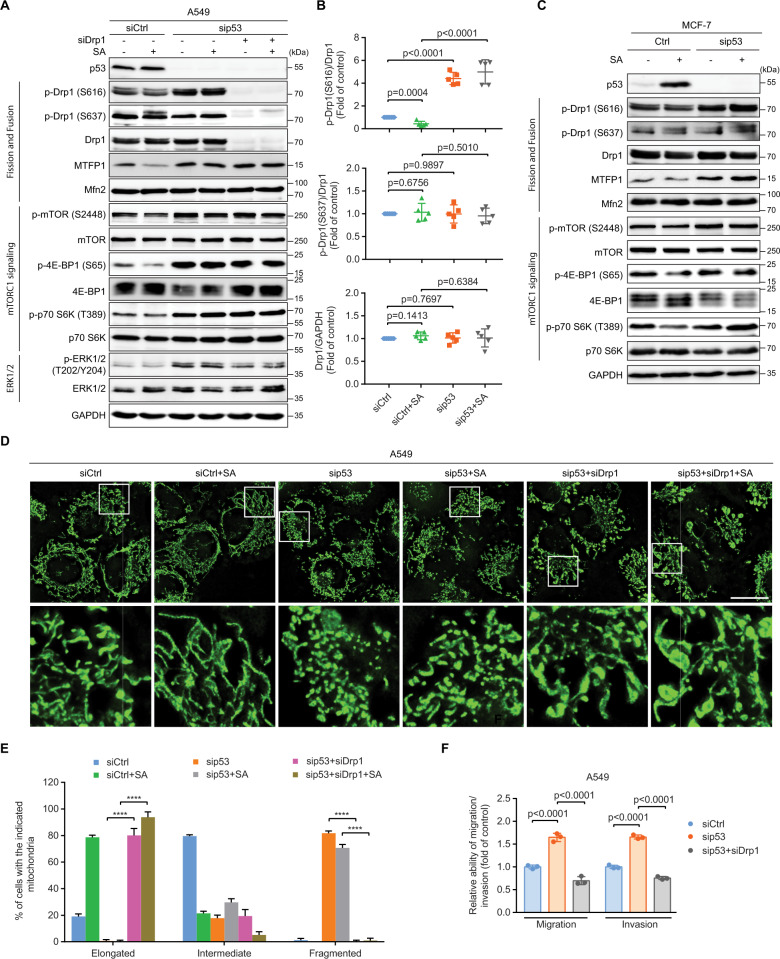
p53 alleviates Drp1-mediated mitochondrial fission and thereby restrains cell migration and invasion. **A** Immunoblot of the indicated proteins in siCtrl, sip53, and p53/Drp1 double-knockdown (sip53 + siDrp1) A549 cells with and without 20 µM SA treatment for 24 h. GAPDH was used as a loading control. **B** Quantification of levels of Drp1, p-Drp1 (S637), and p-Drp1 (S616) in Fig. 4 A. **C** Immunoblot of the indicated proteins in siCtrl and sip53 MCF-7 cells with and without 20 µM SA treatment for 24 h. GAPDH was used as a loading control. Representative images (**D**) and quantification (**E**) of mitochondrial morphology in siCtrl (*n* = 226), siCtrl+SA (*n* = 232), sip53 (*n* = 205), sip53+SA (*n* = 214), sip53 + siDrp1 (*n* = 230), and sip53 + siDrp1+SA (*n* = 214) A549 cells. Boxed regions in (**D**) are shown enlarged in the bottom panels. Scale bar: 20 µm. **F** Transwell assays for siCtrl, sip53, and sip53 + siDrp1 A549 cells. Error bars represent mean ± SD. Data were analyzed by two-tailed unpaired Student’s *t* test (**B**) or one-way ANOVA with Tukey’s multiple comparisons test (**E** and **F**). ****p < 0.0001.

To delineate the underlying molecular mechanism responsible for enhanced mitochondrial fragmentation during p53 loss, we investigated changes in the expression and phosphorylation of mitochondrial fusion and fission factors in response to changes in p53 levels. The mRNA levels of mitochondrial fusion and fission factors were unaltered upon p53 silencing in A549 cells (Fig. S3A). Additionally, both SA-induced p53 upregulation and siRNA-mediated p53 knockdown did not affect the protein levels of total Drp1 and Mfn2 (Fig. 4A).

Phosphorylation of serine 616 (S616) on Drp1 enables Drp1-directed mitochondrial fission, whereas phosphorylation of serine 637 (S637) on the same protein abolishes its GTPase activity and inhibits the fission of mitochondria [35]. While Drp1 S637 phosphorylation was unaffected by the expression levels of p53, SA-elevated p53 expression that caused mitochondrial elongation (Fig. 3B, C) triggered a more than 50% reduction in the pro-fission S616 phosphorylation of Drp1 (Fig. 4A, B). Conversely, in both untreated and SA-treated p53-silenced cells whose mitochondria were extensively fragmented (Fig. 3B, C), Drp1 S616 phosphorylation was increased by over 4-fold when compared to that of the p53 WT controls (Fig. 4A, B). These results suggest that p53 might control mitochondrial morphology by modulating the phosphorylation of S616 on Drp1.

Reportedly, mTORC1 phosphorylates 4E-BPs to enable MTFP1 translation, thereby stimulating Drp1-mediated mitochondrial fission [7]. In agreement with the Drp1 S616 phosphorylation and mitochondrial fission activity, the levels of MTFP1 and proteins relevant to mTORC1 signaling, including the phosphorylations of mTOR S2448, 4E-BP1 S65, and S6K1 (p70 S6K) T389 were decreased following SA-stimulated endogenous p53 expression in A549 cells. p53 depletion enhanced MTFP1 protein levels and mTORC1 activity in both untreated and SA-treated cells (Fig. 4A). Furthermore, elevated p53 expression by SA also hampered the T202/Y204 phosphorylation of ERK1/2 (Fig. 4A) whose activation was governed by Drp1-mediated mitochondrial fission [2]. p53 silencing, however, augmented ERK1/2 phosphorylation in both untreated and SA-treated cells (Fig. 4A).

To verify the regulation of mitochondrial dynamics and mTORC1 signaling by p53 is not restricted to A549 cells, we examined mitochondrial fission and fusion factors and mTORC1 signaling in MCF-7 (Fig. 4C) and H1299 (Fig. S3B, C) cells. Similar to findings in A549 cells, both SA-induced endogenous p53 upregulation and siRNA-mediated knockdown of p53 had no significant effect on the levels of Drp1, Mfn2, and Drp1 S637 phosphorylation in MCF-7 cells. Conversely, there was a robust increase in Drp1 S616 phosphorylation, correlated with an elevation of MTFP1 protein levels and mTOR, 4E-BP1, and S6K1 phosphorylations in both untreated and SA-treated MCF-7 cells upon p53 silencing (Fig. 4C). In sharp contrast to p53 WT A549 and MCF-7 cells, SA treatment did not affect the levels of Drp1, Mfn2, and Drp1 S637 phosphorylation, but it induced an ~2.5-fold increase in the levels of Drp1 S616 phosphorylation and a corresponding upregulation of MTFP1 protein levels and mTOR, 4E-BP1, and S6K1 phosphorylations in p53-null H1299 cells (Fig. S3B, C). Importantly, overexpression of exogenous WT p53 in control and SA-treated H1299 cells diminished Drp1 S616 phosphorylation by more than 40% and 50%, respectively, whereas levels of total Drp1, Mfn2, and Drp1 S637 phosphorylation were unaffected (Fig. S3B, C). In line with this, phosphorylations of mTOR, 4E-BP1, and S6K1 and the protein levels of MTFP1 were decreased upon exogenous expression of WT p53 in both untreated and SA-treated H1299 cells (Fig. S3B). Altogether, these results support the notion that p53 drives mitochondrial elongation by inhibiting the phosphorylation of S616 on Drp1, accompanied by reducing MTFP1 protein levels and the mTORC1 activity.

To further investigate whether Drp1-mediated mitochondrial fission might contribute to the metastatic phenotype driven by p53 loss, we performed a double-knockdown of both p53 and Drp1 in A549 cells. In line with our conjecture that mTORC1-controlled MTFP1 protein translation is the upstream signaling that modulates Drp1 activity and mitochondrial fission [7], co-knockdown of Drp1 in p53-silenced cells did not affect the total amounts and phosphorylations of mTOR, 4E-BP1, and S6K1 and the protein levels of MTFP1 as compared to those in cells with p53 knockdown alone in either the absence or presence of SA (Fig. 4A). In contrast, ERK1/2 T202/Y204 phosphorylation was reduced in cells with p53/Drp1 double-knockdown when compared to that in cells with p53 knockdown alone (Fig. 4A), suggesting that ERK1/2 might be the downstream signaling regulated by Drp1-driven mitochondrial fission. Significantly, Drp1 depletion not only rescued p53 deficiency-induced mitochondrial fragmentation but also exaggerated mitochondrial elongation. Over 80% and 90% of mitochondria were elongated in untreated and SA-treated p53/Drp1 double-knockdown cells, respectively (as compared to < 1% in untreated and SA-treated p53-depleted cells) (Fig. 4D, E). Most notably, Drp1 depletion abolished accelerated cell migration in both untreated and SA-treated p53 knockdown cells (Fig. S3D-F). Consistently, transwell assays showed a more than 2-fold decrease in the migratory and invasive abilities of p53/Drp1 double-knockdown cells, when compared to those with only p53-knockdown (Fig. 4F). Taken together, these results demonstrate that elevated mitochondrial fragmentation caused by increased Drp1 S616 phosphorylation is responsible for the aggressive cell migration and invasion seen upon p53 loss.

### p53 diminishes mTORC1-controlled MTFP1 protein levels to attenuate Drp1-driven mitochondrial fission and invasive cell migration

**Fig. 5:**
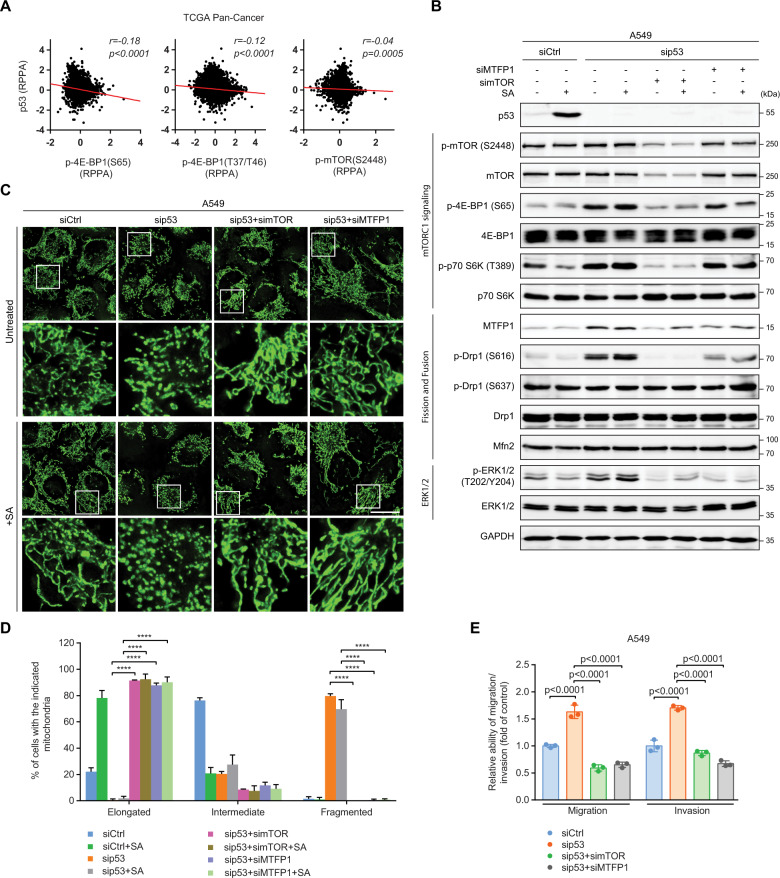
p53 diminishes mTORC1-controlled MTFP1 protein levels to attenuate Drp1-driven mitochondrial fission and invasive cell migration. **A** Correlations between the RPPA levels of p53 and the indicated proteins (*n* = 7694 samples). Data were extracted from TCGA. **B** Immunoblot of the indicated proteins in siCtrl, sip53, p53/mTOR double-knockdown (sip53+simTOR), and p53/MTFP1 double-knockdown (sip53 + siMTFP1) A549 cells with and without 20 µM SA treatment for 24 h. GAPDH was used as a loading control. Representative images (**C**) and quantification (**D**) of mitochondrial morphology in siCtrl (*n* = 203), siCtrl+SA (*n* = 207), sip53 (*n* = 200), sip53+SA (*n* = 213), sip53+simTOR (*n* = 234), sip53+simTOR+SA (*n* = 209), sip53 + siMTFP1 (*n* = 214), and sip53 + siMTFP1+SA (*n* = 213) A549 cells. Boxed regions in (**C**) are shown enlarged in the bottom panels of each group. Scale bar: 20 µm. **E** Transwell assays for siCtrl, sip53, sip53+simTOR, and sip53 + siMTFP1 A549 cells. Error bars represent mean ± SD. Data were analyzed by one-way ANOVA with Tukey’s multiple comparisons test. ****p < 0.0001.

Our results suggest that mTORC1 signaling might function upstream of Drp1 to drive Drp1 S616 phosphorylation. We next examined the relationship between p53 and mTORC1. Pan-Cancer database analysis showed that p53 protein levels correlated inversely with 4E-BP1 S65, 4E-BP1 T37/T46, and mTOR S2448 phosphorylations (Fig. 5A), suggesting a negative effect of p53 on mTORC1 activity. Since p53 acts predominantly as a transcription factor, we examined whether the transcriptional regulatory function of p53 is involved in the p53-driven suppression of mTORC1 activity and Drp1 S616 phosphorylation. The p53-specific transcriptional inhibitor, pifithrin-α (PFT-α), successfully suppressed the induction of the p53 downstream target p21 under the condition of SA-induced p53 upregulation. Interestingly, PFT-α did not affect p53 protein levels, but strongly enhanced mTOR, 4E-BP1, and Drp1 S616 phosphorylations and MTFP1 protein expression in both control and SA-treated A549 cells (Fig. S4A). These results are in line with the observations using p53 gene knockdown, suggesting that the transcriptional activity of p53 is essential for p53-mediated inhibition of mTORC1 and Drp1 activities. Remarkably, p53 silencing attenuated the gene expression of negative regulators of mTOR signaling, including PTEN, AMPKβ1, Sestrin1/2, and TSC2 (Fig. S4B).

Increased or decreased p53 expression did not alter MTFP1 mRNA levels (Fig. S4C), suggesting that MTFP1 expression was controlled at the protein level. To experimentally verify the interplay and chronology of mTORC1, MTFP1, and Drp1, we performed double knockdowns of p53 and mTOR or p53 and MTFP1 in A549 cells (Fig. 5B). mTOR knockdown reduced the phosphorylations of mTORC1 downstream effectors 4E-BP1 and S6K1 and diminished MTFP1 protein levels in both untreated and SA-treated p53-silenced A549 cells. In contrast, MTFP1 knockdown did not affect the total amounts and phosphorylations of mTORC1-relevant factors. The mTOR or MTFP1 silencing successfully abolished increased Drp1 S616 phosphorylation in both untreated and SA-treated p53-depleted cells, while both mTOR and MTFP1 knockdown did not affect total Drp1 and Mfn2 protein levels (Fig. 5B). In line with the reduction of ERK1/2 activity seen upon Drp1 silencing (Fig. 4A), ERK1/2 phosphorylation was diminished in both untreated and SA-treated p53/mTOR or p53/MTFP1 double-knockdown cells when compared with similar cells with only p53 silencing (Fig. 5B). These results provide compelling evidence that mTORC1 mediating MTFP1 protein expression is required for the increased Drp1 S616 phosphorylation during p53 loss.

We further unraveled the contribution of the mTORC1/MTFP1 axis in modulating mitochondrial dynamics. Knockdown of either mTOR or MTFP1 completely rescued fragmented mitochondria in both untreated and SA-treated p53-depleted cells (Fig. 5C, D). Mitochondria were elongated in ~90% of p53/mTOR or p53/MTFP1 double-knockdown cells untreated or treated with SA (Fig. 5D). Consistent with the finding that mitochondrial dynamics impacts cell migration and invasion, exaggerated mitochondrial elongation triggered by mTOR or MTFP1 knockdown fully suppressed the migratory capacity of both untreated and SA-treated p53-depleted cells (Fig. S4D, E). Transwell assays also showed that mTOR or MTFP1 depletion completely abrogated the increases in cell migration and invasion caused by p53 loss (Fig. 5E). These results demonstrate that the control of MTFP1 protein levels by mTORC1 signaling is critical for p53 deficiency-induced mitochondrial fragmentation and accelerated cell migration and invasion.

### Activation of mTORC1/MTFP1/Drp1/ERK1/2 signaling axis is required for the EMT switch, MMP9 elevation, and cancer dissemination upon WT p53 loss

**Fig. 6:**
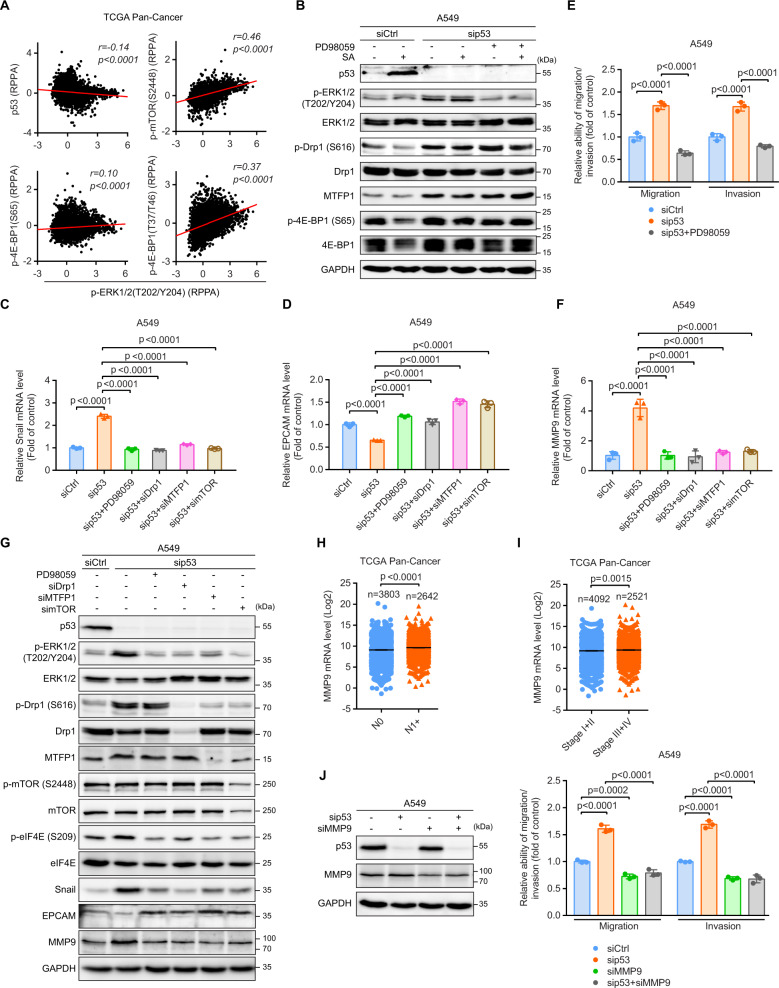
Activation of mTORC1/MTFP1/Drp1/ERK1/2 signaling axis is required for the EMT switch, MMP9 elevation, and cancer dissemination upon WT p53 loss. **A** Correlations between the RPPA levels of p-ERK1/2 (T202/Y204) and the indicated proteins (*n* = 7694 samples). Data were extracted from TCGA. **B** Immunoblot of the indicated proteins in siCtrl, sip53, and PD98059-treated sip53 (sip53 + PD98059) A549 cells with and without 20 µM SA treatment for 24 h. GAPDH was used as a loading control. qRT-PCR analysis of the mRNA levels of (**C**) Snail and (**D**) EPCAM in siCtrl, sip53, sip53 + PD98059, sip53 + siDrp1, sip53 + siMTFP1, and sip53+simTOR A549 cells. **E** Transwell assays for siCtrl, sip53, and sip53 + PD98059 A549 cells. **F** qRT-PCR analysis of MMP9 mRNA expression in siCtrl, sip53, sip53 + PD98059, sip53 + siDrp1, sip53 + siMTFP1, and sip53+simTOR A549 cells. **G** Immunoblot of the indicated proteins in siCtrl, sip53, sip53 + PD98059, sip53 + siDrp1, sip53 + siMTFP1, and sip53+simTOR A549 cells. GAPDH was used as a loading control. MMP9 mRNA expression in (**H**) N0 and N1 + or (**I**) stage I + II and III + IV tumors. Data were extracted from TCGA. **J** Transwell assays for siCtrl, sip53, MMP9-silenced (siMMP9), and p53/MMP9 double-knockdown (sip53 + siMMP9) A549 cells. Error bars represent mean ± SD (**C**–**F** and **J**) or SEM (**H** and **I**). Data were analyzed by one-way ANOVA with Tukey’s multiple comparisons test (**C**–**F** and **J**) or two-tailed unpaired Student’s *t* test (**H** and **I**).

Building on our findings that p53 silencing elevated ERK1/2 phosphorylation but could be counteracted by co-knockdown of Drp1, mTOR, or MTFP1 (Figs. 4A and 5B), we theorized that mitochondrial fission governed by mTORC1/MTFP1/Drp1 axis might activate ERK1/2 signaling to direct cell migration and invasion upon p53 loss. Indeed, we observed that ERK1/2 phosphorylation displayed inverse and direct correlations with p53 and the phosphorylations of mTORC1-relevant factors (mTOR and 4E-BP1), respectively (Fig. 6A). Inhibition of ERK1/2 with PD98059 had no significant effect on MTFP1 protein levels and the phosphorylations of Drp1 and 4E-BP1 in p53-silenced cells with and without SA-treatment (Fig. 6B). In contrast, similar to observations seen upon depletion of Drp1, MTFP1, or mTOR, inhibition of ERK1/2 rescued the aggressive cell phenotype caused by p53 silencing (Fig. S5A), diminished the mRNA levels of the EMT-promoting transcription factor Snail (Fig. 6C), and restored EPCAM mRNA expression (Fig. 6D). ERK1/2 inhibition also hampered p53 deficiency-accelerated cell motility and invasion (Figs. 6E and S5B, C). Thus, ERK1/2 signaling functions downstream of mitochondria whose morphology is modulated by the mTORC1/MTFP1/Drp1 axis to favor cell dissemination upon p53 loss.

Several studies have highlighted that ERK1/2 activation stimulates the expression of MMP9, which contributes to the proteolytic degradation of the extracellular matrix [36–40]. Strikingly, p53 silencing induced approximately 4- and 4.4-fold increases in MMP9 mRNA levels in A549 (Fig. 6F) and MCF-7 (Fig. S5D) cells, respectively. Inhibition of ERK1/2 or co-knockdown of Drp1, MTFP1, or mTOR completely abolished increased MMP9 gene expression in p53-depleted cells (Figs. 6F and S5D). Consistently, p53 silencing elevated MMP9 and Snail along with repressed EPCAM protein levels but these effects could be counteracted by inhibition of ERK1/2 or co-knockdown of Drp1, MTFP1, or mTOR (Fig. 6G). Reportedly, ERK1/2 stimulates MMP9 mRNA translation by regulating the phosphorylation of S209 on eIF4E [37, 41]. In agreement with MMP9, we showed that p53 depletion augmented eIF4E S209 phosphorylation, which was abolished by inhibiting the activation of the mTORC1/MTFP1/Drp1/ERK1/2 axis (Fig. 6G), suggesting that MMP9 may also be translationally controlled by ERK1/2-mediated eIF4E phosphorylation. Moreover, a comparison of differential gene expression between tumors harboring WT and MUT *TP53* revealed that MMP9 mRNA levels were lower in patients having WT *TP53* than in those with MUT *TP53* (Fig. S5E). p53 protein levels also exerted a negative correlation with MMP9 mRNA levels in cancers having WT *TP53* (Fig. S5F). Collectively, these results indicate that p53 diminishes MMP9 expression and the EMT phenotypic transition by inhibiting the mTORC1/MTFP1/Drp1/ERK1/2 signaling axis.

Consistent with previous literature showing that increased MMP9 expression promotes cancer cell migration, invasion, and metastasis [42], MMP9 mRNA levels were enhanced in N1 + (Fig. 6H) and advanced-stage (III + IV) (Fig. 6I) tumors when compared to those in N0 and the earlier-stage (I + II) tumors, respectively. MMP9 mRNA levels were also higher in metastatic than in primary melanoma (Fig. S5G). Higher MMP9 levels were associated with worse overall survival in cancer patients (Fig. S5H, left). The median overall survival was 86.14 and 93.20 months in patients expressing high and low MMP9 mRNA levels, respectively. Of cancer patients harboring WT *TP53*, those with high MMP9 mRNA and low p53 protein expression showed a significantly increased risk of death when compared to those with low MMP9 mRNA and high p53 protein expression (the median overall survival was 112.1 and 146.1 months, respectively) (Fig. S5H, right). These results corroborate the clinical implications for MMP9 in cancers.

Finally, we sought to examine the regulatory role of MMP9 in the metastatic phenotype of p53-silenced cells by performing a p53/MMP9 double-knockdown in A549 cells (Fig. 6J). MMP9 knockdown reduced both the migratory and invasive abilities of p53 WT cells by roughly 30%. More important, MMP9 knockdown completely abolished increased cell migration and invasion caused by p53 silencing (Fig. 6J). These results indicate that MMP9 is indispensable for the metastatic dissemination of cancer cells upon p53 loss.

## Discussion

This study unravels a molecular explanation of how tumor suppressor p53 modulates mitochondrial dynamics to restrict cancer cell dissemination. For the first time, we link p53 to mitochondria-dependent regulation of malignant properties of cancers, including cell motility and metastasis. p53 is canonically known to suppress cancer development by regulating multiple cell fate-determining genes involved in cell cycle arrest, DNA damage repair, senescence, and apoptosis [43, 44]. p53 also constrains the metastatic abilities of cancer cells by transcriptionally controlling components of the metastatic cascade [24]. We present evidence here that p53 suppresses cancer dissemination via mitochondrial dynamics modulation and provide an alternative mechanism that coordinates aggressive phenotypes in cancers harboring compromised p53. The strong positive correlation between p53 expression levels and overall survival in cancer patients may be at least in part resulted from its anti-metastatic effects.

*TP53* is the most frequently altered gene in human cancer [45]. More than one-half of human tumors harbor mutations in the *TP53* gene [46, 47], with approximately 75% of these mutations being missense-type at hot spots [29–31]. In line with previous literature showing that MUT p53 accelerates cancer cell motility, invasion, and metastasis [32, 43], our examination of the TCGA Pan-Cancer cohort observes a greater fraction of cancer patients developing metastases to lymph nodes or distant organs in the MUT *TP53* group relative to the WT *TP53* group. Critically, patients carrying *TP53* missense mutations have a higher incidence of lymph node metastases than their *TP53* gene deletion counterparts, suggesting that *TP53* missense mutations possess gain-of-function mechanisms mediating cancer cell dissemination. Reportedly, several gain-of-function MUT p53s, such as P151S, R175H, G245C, and R282W, can inhibit the activation of AMP-activated protein kinase (AMPK) [48], an upstream negative regulator of mTORC1 signaling [8], through directly binding to the AMPKα subunit under conditions of energy stress, thus resulting in increased activation of the mTOR/S6 ribosomal protein pathway and cell migration [48]. *TP53* missense mutations also play a gain-of-function role in stimulating mTORC1 activation by favoring the interaction between mTOR and Rheb [49], an upstream positive regulator of mTOR activity [8]. We demonstrate here that mTORC1 accelerates cancer cell migration and invasion by enhancing Drp1-governed mitochondrial fission. These discoveries raise the possible links among MUT p53, mTORC1, cancer metastasis, and mitochondrial dynamics. Further work examining whether missense mutations in the *TP53* gene affect mitochondrial morphology to promote invasive cell migration may provide an additional molecular explanation for the pro-metastatic gain-of-function activities of MUT p53.

mTORC1 is commonly hyper-activated in p53-compromised cancers [19–23] and contributes to invasive cell migration [13–15]. However, the underlying molecular signaling pathways behind the pro-migratory activity of mTOR remain poorly understood. We show here that mTORC1 accelerates cancer dissemination by directing MTFP1 protein expression. MTFP1 then facilitates Drp1 S616 phosphorylation and mitochondrial fission upon p53 loss. These findings provide an important insight into a hitherto unknown mechanism that links mTORC1 to cancer metastasis. Recently, MTFP1 has also been implicated in promoting the migratory ability of adipose-derived stem cells [50] as well as MMP9 expression and cancer metastasis in hepatocellular carcinoma [51] although the underlying mechanisms are still unknown. MTFP1 is an integral protein of the mitochondrial inner membrane and contributes to mitochondrial fission dependent on Drp1 [7, 11, 12]. Additionally, Drp1-mediated mitochondrial fission enhances the metastatic abilities of cancer cells via multiple mechanisms [2–5]. Increased Drp1-dependent mitochondrial fission activates ERK1/2 signaling [2], which acts as a positive regulatory pathway upstream of the metastatic driver MMP9 [36–40]. Strikingly, MMP9 expression is enhanced in the context of exaggerated cell invasion and mitochondrial fragmentation caused by increased Drp1 S616 phosphorylation [52]. Thus, MTFP1 may regulate MMP9 expression and invasive cell migration indirectly through its ability to promote Drp1-dependent mitochondrial fission and ERK1/2 activation. In line with this premise, our results show that enhanced MTFP1 protein expression upon p53 loss heightens Drp1 S616 phosphorylation and mitochondrial fission to enable ERK1/2 activation, resulting in the EMT-associated morphologic switch, elevated MMP9 expression, and cancer cell dissemination. This underscores components of the mTORC1/MTFP1/Drp1/ERK1/2 signaling as potential and effective therapeutic targets for treating malignant and metastatic p53-compromised tumors [15, 53].

It is interesting to note that enhanced mitochondrial fragmentation is not always due to the accumulation of damaged mitochondria. Instead, mitochondrial biogenesis, by which new functional mitochondria are generated, also requires the initiation of Drp1-driven mitochondrial fission [54–56]. Fissions derived from mitochondrial dysfunction are associated with increased mitochondrial ROS and diminished ΔΨm [55, 57, 58], whereas the mitochondrial integrity during fissions in the biogenesis of new mitochondria remains unchanged [55]. Intriguingly, our data show that depletion of p53 exaggerates mitochondrial fragmentation, but it does not affect ΔΨm and mitochondrial ROS levels. In addition, it has been reported that mitochondrial biogenesis is regulated by the mTORC1/4E-BP pathway which stimulates the translation of mRNAs encoding mitochondria-related proteins [59]. Here, we show that p53 depletion activates mTORC1/4E-BP1 signaling that regulates MTFP1 protein expression to govern Drp1-mediated mitochondrial fission. Thus, we speculate that increased mitochondrial fission upon p53 loss is associated with stimulation of mitochondrial biogenesis, but not accumulation of damaged mitochondria. This would explain how the mitochondrial integrity remains constant in the context of p53 deficiency-induced mitochondrial fragmentation.

Accumulating evidence illustrates the critical roles of intracellular calcium (Ca^2+^) signaling in the regulation of key steps of the metastatic cascade, including EMT, focal adhesion turnover, lamellipodia formation, and the degradation of the extracellular matrix [60–62]. Notably, mitochondrial fission reduces the potential of endoplasmic reticulum (ER)-mitochondrial contacts and thereby attenuates the capacity of mitochondria to sequester Ca^2+^ released from the ER, leading to an increase in cytosolic Ca^2+^ levels [63, 64]. Moreover, mitochondrial fission resulting in elevated Ca^2+^ levels in the cytoplasm activates multiple Ca^2+^-dependent pathways regulating cellular behaviors, including cell migration and invasion [2, 65, 66]. Consistently, we find that exaggerated mitochondrial fission upon p53 loss triggers increased phosphorylation of ERK1/2, which is a downstream target of Ca^2+^/calmodulin-dependent protein kinase II (CaMKII), a major decoder of the intracellular Ca^2+^ oscillations [2, 67]. Pharmacological inhibition of ERK1/2 activity highlights an indispensable role of ERK1/2 signaling in controlling EMT, MMP9 expression, and the metastatic abilities of cancer cells upon p53 loss. Our observations are supported by studies indicating that ERK1/2 stimulates MMP9 gene expression via regulating the activity of the transcription factors NF-ĸB (nuclear factor kappa-light-chain-enhancer of activated B cells) and AP-1 (activator protein-1) [38–40]. In agreement with previous findings that ERK1/2 stimulates MMP9 mRNA translation by directing the phosphorylation of S209 on eIF4E [37, 41], we also observe the concomitant ERK1/2-dependent increases in both MMP9 protein expression and eIF4E S209 phosphorylation in p53-depleted cells. Furthermore, ERK1/2 signaling is implicated in controlling numerous other components of the cell motility machinery [68, 69]. For example, ERK1/2 signaling promotes cancer cell migration, invasion, and EMT by mediating the expression or the transcriptional activity of EMT-inducing transcription factors Twist1 [70, 71], Snail [72, 73], and Slug [74]. Thus, specific ERK1/2 inhibition may be beneficial to slow cancer metastasis in patients harboring compromised p53.

**Fig. 7:**
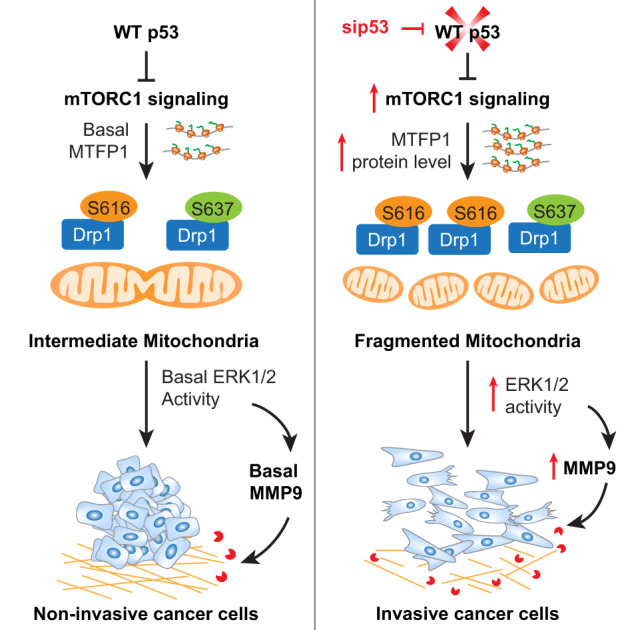
Schematic model of how p53 modulates mitochondrial dynamics to constrain EMT, MMP9 expression, and invasive cell migration. WT p53 suppresses mTORC1-directed MTFP1 protein expression and the aberrant phosphorylation of Drp1 at the pro-fission site S616, maintaining the predominantly intermediate state of mitochondria, and thereby constraining ERK1/2-mediated cell migration and invasion. Loss of WT p53 elevates mTORC1 activity, MTFP1 protein levels, and the phosphorylation of S616 on Drp1, shifting mitochondrial dynamics toward fission to promote ERK1/2 activation and resulting in EMT-like changes in cell morphology, increased MMP9 expression, and cell dissemination.

In summary, we have illustrated how p53 can modulate mitochondrial dynamics via controlling the mTORC1/MTFP1/Drp1 axis to restrict cancer cell dissemination (Fig. 7). The molecular mechanism uncovered in this study is likely a general phenomenon. Indeed, we could observe the downregulation of WT p53 and the elevations of the pro-fission factor Drp1 and the metastatic driver MMP9 in aggressive and malignant tumors across cancers in The Cancer Genome Atlas. Additionally, Pan-Cancer analysis also showed that reduced WT p53 expression and enhanced expression of Drp1 or MMP9 are strongly correlated with poor prognosis. Our results offer a new molecular explanation for the aggressive malignant phenotypes of p53-compromised cancers and suggest that targeting mitochondrial fission and its downstream ERK1/2 signaling pathway may diminish the spread of p53-compromised cancer cells.

## Materials and methods

Further details for all experimental procedures, reagents, and treatments are provided in Supplementary Materials and Methods.

### Cell culture

A549, H1299, and MCF-7 cells were cultured at 37 °C in a humidified incubator supplemented with 5% CO_2_ as described in Supplementary Materials and Methods.

### siRNA and plasmid transfection

Transfection of siRNAs and the pcDNA3 p53 WT plasmid were performed using Lipofectamine RNAimax and Lipofectamine 2000 reagents (Invitrogen), respectively, according to the manufacturer’s protocols as described in Supplementary Materials and Methods. siRNA sequences are listed in Supplementary Table S1.

### Immunoblotting and qRT-PCR

Immunoblotting and qRT-PCR were performed according to our previous works [75, 76], with some modifications. Detailed procedures are described in Supplementary Materials and Methods. Primers used for qRT-PCR are listed in Supplementary Table S2.

### Live single-cell tracking

The single-cell motility assay was performed as described previously [77].

### In vitro wound-healing assay

The scratch wound-healing assay was performed as previously described [78].

### Transwell cell migration and invasion assays

Transwell cell migration and invasion assays were performed using 24-well cell culture inserts (Corning) based on the manufacturer’s recommendations and previous work [79], with some modifications. Detailed procedures are described in Supplementary Materials and Methods.

### Live-cell fluorescence microscopy and quantification of mitochondrial morphology

Live-cell fluorescence images were acquired on a Nikon Eclipse Ti inverted microscope after cells were stained with MitoTracker Green FM (Invitrogen) according to the manufacturer’s protocol as described in Supplementary Materials and Methods.

### Software and statistical analysis

All analyzes of clinical data carried out in this paper are based upon data generated by The Cancer Genome Atlas (TCGA) Research Network (https://www.cancer.gov/tcga) except the association between the presence of *TP53* mutations versus the probability of distant metastasis (MSK-IMPACT cohort) (Fig. 1B). Data were presented as means ± SD or SEM of at least three independent experiments. All graphing and statistical analyses were performed using GraphPad Prism 7 software. For all in vitro experiments, the samples were randomly allocated into different experimental groups based on the relevant treatments. All microscopic images were randomly taken from different areas of a cell culture dish or chamber. Details of sample size (n), statistical test, and *p-value* applied for each experiment were indicated in the figure legends. Values with *p* < 0.05 and designated with * are considered statistically significant. All composite figures were assembled in Adobe Illustrator.

## Supplementary information

Supplementary Information

## Data Availability

All data generated or analyzed during this study are available from the corresponding authors on reasonable request.
