# Supplementary material for: Tumor suppressor p53 restrains cancer cell dissemination by modulating mitochondrial dynamics
Source: Oncogenesis. 2022 May 19;11(1):26. doi: 10.1038/s41389-022-00401-x (PMC9120037; doi:10.1038/s41389-022-00401-x)
Supplement: Supplementary file 1 — Supplementary Information [file 41389_2022_401_MOESM1_ESM.pdf]

Supplementary Information for

Tumor Suppressor p53 Restrains Cancer Cell Dissemination by Modulating Mitochondrial Dynamics

Trinh T. T. Phan[1,*], Yu-Chun Lin[2], Yu-Ting Chou[3], Chien-Wei Wu[1], and Lih-Yuan Lin[1,*]

[1]Institute of Molecular and Cellular Biology, College of Life Science, National Tsing Hua University, Hsinchu 300044, Taiwan R.O.C

[2]Institute of Molecular Medicine, College of Life Science, National Tsing Hua University, Hsinchu 300044, Taiwan R.O.C

[3]Institute of Biotechnology, College of Life Science, National Tsing Hua University, Hsinchu 300044, Taiwan R.O.C

***Corresponding authors:** Lih-Yuan Lin, Trinh T. T. Phan

Email: lylin@life.nthu.edu.tw (LYL), s107080893@m107.nthu.edu.tw (TTTP)

This PDF file includes:

Supplementary Materials and Methods

References for Supplementary Information

Supplementary Figure Legends

Supplementary Figures S1 to S5

Supplementary Tables S1 and S2

Supplementary Materials and Methods

Cell lines and cell culture conditions

A549 and H1299 cells were maintained in Roswell Park Memorial Institute (RPMI) 1640 medium supplemented with 10% heat-inactivated fetal bovine serum (FBS), 0.22% sodium bicarbonate, 2 mM L-glutamine (L-Gln), and 100 units/ml penicillin/streptomycin (P/S). MCF-7 cells were maintained in Dulbecco's modified Eagle's medium (DMEM) containing 10% FBS, 0.37% sodium bicarbonate, 2 mM L-Gln, and 100 units/ml P/S. All cells were cultured at 37 °C in a humidified incubator supplemented with 5% CO_2. A549, H1299, and MCF-7 cells were obtained from the American Type Culture Collection (ATCC, Manassas, VA, USA).

Reagents and treatments

Reagents for cell cultures were purchased from Invitrogen Gibco (Grand Island, NY, USA). Other chemicals in this study were purchased from Sigma-Aldrich (St. Louis, MO, USA) unless specified. Sodium arsenite was obtained from Merck (Darmstadt, Germany). MTT (3-(4,5-dimethylthiazol-2-yl-2, 5-diphenyl tetrazolium bromide)) was purchased from Alfa Aesar (Thermo Fisher Scientific, Leicestershire, UK). MitoTracker Green FM, MitoSOX Red, JC-1 dye, TRIzol reagent, and reagents for reverse transcription and transfection were purchased from Invitrogen (Carlsbad, CA, USA). p53, Drp1, mTOR, MTFP1, and Stealth RNAi siRNA Negative Control siRNAs were purchased from Invitrogen (Carlsbad, CA, USA). ON-TARGETplus SMARTpool siRNAs specific for human MMP9 and ON-TARGETplus Non-targeting Pool control siRNAs were purchased from Dharmacon (Lafayette, CO, USA). Matrigel basement membrane matrix was purchased from Corning (Tewksbury, MA, USA). Primers used in qRT-PCR were purchased from Integrated DNA Technologies (Coralville, IA, USA). PD98059 was purchased from Enzo Life Sciences (Farmingdale, NY, USA).

Sodium arsenite (SA) (Merck) was dissolved in deionized water and added to culture medium at indicated concentrations (10, 20, 40, and 80 μM) for 24 h. Pifithrin-α (PFT-α) (Sigma-Aldrich) and PD98059 (Enzo Life Sciences) were dissolved in dimethyl sulfoxide (DMSO) (Sigma-Aldrich). Cells were pretreated with PFT-α or PD98059 at 20 μM for 3 h or 30 μM for 2 h, respectively, prior to treatment with 20 μM SA for 24 h.

siRNA and plasmid transfection

Small interference (si)RNA-mediated gene knockdown experiments were carried out with cells transfected with 10 nM (Invitrogen) or 20 nM (Dharmacon) siRNA for 3 days using Lipofectamine RNAimax (Invitrogen) following the manufacturer's reverse transfection instructions. A Stealth RNAi siRNA Negative Control (Invitrogen) or ON-TARGETplus Non-targeting Pool (Dharmacon) was used as control. See Table S1 for information about siRNA target sequences.

The pcDNA3 p53 WT plasmid was constructed by inserting a WT p53 gene (393 amino acids) into a pcDNA3 plasmid. Transient expression of WT p53 in H1299 cells was carried out by transfecting cells with the pcDNA3 p53 WT plasmids for 2 days using Lipofectamine 2000 reagent (Invitrogen) according to the manufacturer's protocol. An empty pcDNA3 plasmid was used as control.

Cell lysis, immunoblotting, and antibodies

Cells were harvested by trypsinization and centrifugation at 700 × *g* for 5 min at 4 °C and lysed in ice-cold radioimmunoprecipitation assay (RIPA) lysis buffer (50 mM Tris-HCl (pH 8.0), 150 mM NaCl, 5 mM EDTA (pH 8.0), 1% NP-40, 0.1% SDS, and 0.5% sodium deoxycholate) supplemented with complete protease and phosphatase inhibitor cocktails (Fivephoton Biochemicals, San Diego, CA, USA). Cell suspension was subsequently incubated on ice for 10 min and then vortexed vigorously for 5 sec. The incubation and vortexing steps were repeated

four times, and cell lysates were separated from debris by centrifugation at 16 000 × *g* for 20 min at 4 °C. After centrifugation, the supernatants were transferred to new tubes and protein concentrations were determined using the Bio-Rad protein assay (Bio-Rad, Hercules, CA, USA). Proteins in cell lysates were separated on SDS-PAGE and then transferred electrophoretically onto PVDF membranes (GE Healthcare, Milwaukee, WI, USA) using a transfer cell (Bio-Rad, Hercules, CA, USA). Membranes were subsequently pre-hybridized in TBST buffer (150 mM NaCl, 10 mM Tris-HCl (pH 8.0), 0.1% Tween-20) with 5% skim milk for 1 h before incubating overnight with appropriate primary antibodies diluted in TBST buffer containing 5% bovine serum albumin (BSA). Antibodies against p53 (GTX70214), GAPDH (GTX100118), Snail (GTX125918), EPCAM (GTX113091), and MMP9 (GTX100458) were purchased from GeneTex (Hsinchu, Taiwan). Antibodies against Drp1 (8570), phospho-Drp1 (S616) (3455), phospho-Drp1 (S637) (4867), Mfn2 (11925), 4E-BP1 (9644), phospho-4E-BP1 (S65) (9456), p70 S6K (2708), phospho-p70 S6K (T389) (9234), mTOR (2972), phospho-mTOR (S2448) (2971), ERK1/2 (9102), phospho-ERK1/2 (T202/Y204) (4370), eIF4E (2067), and phospho-eIF4E (S209) (9741) were purchase from Cell Signaling Technology (Beverly, MA, USA). Antibodies against MTFP1 (ab198217) were from Abcam (Cambridge, UK). All antibodies were used at a 1:1 000 dilution except anti-GAPDH (1:10 000 dilution) and anti-MTFP1 (1:500 dilution).

After primary antibody incubation, membranes were washed three times for 15 min each with TBST buffer, then incubated for 1 h with the respective horseradish peroxidase (HRP)-conjugated secondary antibodies diluted to 5 000 folds in TBST buffer containing 5% skim milk. HRP-conjugated anti-rabbit IgG (NA934V) and anti-mouse IgG (NA931V) were from Amersham (GE Healthcare, Buckinghamshire, UK). Membranes were then washed three times for 15 min each with TBST buffer and detected by chemiluminescence using CyECL Western Blotting Substrate H (Cyrusbioscience, MDBio, Taipei, Taiwan).

Visualization was processed with an ImageQuant LAS 4000 mini biomolecular imager (GE Healthcare), and the intensities of bands were quantified with the UN-SCAN-IT gel analysis software (version 6.1) (Silk Scientific, Orem, UT, USA). Signal intensities of total proteins were normalized to glyceraldehyde 3-phosphate dehydrogenase (GAPDH). Signal intensities of phosphorylated proteins were calculated by dividing the GAPDH-normalized signal intensity for each phosphorylated protein by the GAPDH-normalized intensity of the corresponding total protein.

RNA isolation and quantitative real-time PCR (qRT-PCR)

Total RNA was extracted using TRIzol reagent (Invitrogen) following the procedures provided by the manufacturer. The extracted RNA was reverse-transcribed with a RevertAid First Strand cDNA Synthesis Kit (Invitrogen). The resulting complementary (c)DNA was used for quantitative real-time PCR (qRT-PCR) using SYBR Green PCR Master Mix (Applied Biosystems, Foster City, CA, USA) on a StepOnePlus Real-Time PCR system (Applied Biosystems). Data were acquired and analyzed using StepOne Software v2.3 (Thermo Fisher Scientific). The expression of GAPDH was determined in each sample and used as reference gene. Expression of target genes was compared on the basis of equivalent GAPDH transcripts using the $2^{-\Delta\Delta Ct}$ method. See Table S2 for information about primer sequences.

Live single-cell tracking

The single-cell motility assay was performed as described previously [1]. Briefly, 1×10^4 cells were plated in 6-well cell culture plates (Corning) and incubated in 5% CO_2 at 37 °C. Time-lapse microscopy was performed using an LS620 Microscope (Lumascope, San Diego, CA, USA). Live-cell images were taken automatically every 10 min over a 24 h period. Single-cell migration distance and trajectory were analyzed at two different X-Y positions using the “Manual Tracking” plugin of the ImageJ software (NIH, Bethesda, MD, USA).

In vitro wound-healing assay

The scratch wound-healing assay was performed as previously described [2]. Briefly, cells were grown to confluence in 6-well cell culture plates (Corning). A linear wound was created by scratching the cell monolayer with a sterile p200 pipette tip. Cells were washed several times before incubating in an FBS-free medium. To monitor the migration of cells back into the wound area, cells were imaged at 0 h and 24 h after scratching using a Dino-Eye Microscope Eyepiece Camera (AnMo Electronics Corporation, New Taipei City, Taiwan) connected to a Nikon TMS-F Inverted Phase Contrast Microscope (Nikon, Tokyo, Japan). The area of the wound was quantified by the “Wound Healing Tool” plugin of the ImageJ software (NIH, Bethesda, MD, USA). The relative migration into the wound was calculated by normalizing the measured wound closure area to the area of the initial wound at the 0 h time point.

Transwell cell migration and invasion assays

Transwell cell migration assays were performed using 24-well cell culture inserts with an 8.0-μm pore size transparent polyethylene terephthalate (PET) membrane (Corning) according to the manufacturer's recommendations and previous work [3], with some modifications. Briefly, 2.5×10^4 cells in 200 μl of serum-free medium were loaded into the upper chamber of each insert. The bottom wells were filled with 750 μl of complete medium containing 10% FBS. The cells were cultured in a humidified incubator at 37 °C supplemented with 5% CO_2. After 16 h, nonmigratory cells on the upper surface of the inserts were carefully removed with cotton swabs. Migrated cells attached to the lower surface of the inserts were fixed with 4% paraformaldehyde (PFA) (Electron Microscopy Sciences, Hatfield, PA, USA) for 2 min, permeabilized with 100% methanol for 20 min, then stained with 0.05% crystal violet (Sigma-Aldrich) for 15 min at room temperature. Migrated cells were observed and imaged using a Dino-Eye Microscope Eyepiece Camera (AnMo Electronics Corporation, New Taipei City, Taiwan) connected to a Nikon TMS-F Inverted Phase

Contrast Microscope (Nikon, Tokyo, Japan). The bound crystal violet was eluted with 33% acetic acid (Mallinckrodt Chemicals, Phillipsburg, NJ, USA) and quantified by measuring the absorbance at 595 nm with a microplate reader (Bio-Rad, Hercules, CA, USA). The relative ability of migration was calculated as folds changed in absorbance of the indicated sample in relation to the control. In the invasion assay, 5×10^4 cells were plated onto the Matrigel-coated inserts and the same procedure as described above was followed.

Live-cell fluorescence microscopy and quantification of mitochondrial morphology

1×10^4 A549 cells were grown in poly(D-lysine)-coated borosilicate glass Lab-Tek 8-well chambers (Thermo Scientific) and stained with MitoTracker Green FM (Invitrogen) (50 nM) for 30 min. After staining, cells were washed three times in prewarmed PBS and replaced with fresh prewarmed medium. Live-cell fluorescence images were acquired on a Nikon Eclipse Ti inverted microscope with a 60X oil objective lens (Nikon) and DS-Qi2 CMOS camera (Nikon) using Nikon element AR software (Nikon). Fluorescence images with 15 stacks of 0.3 μm each were deconvoluted using Huygens Essential Software (Scientific Volume Imaging, Hilversum, North Holland, Netherlands). The maximum intensity projections of images were generated by Nikon element AR software (Nikon). Images were mainly processed and analyzed using Nikon element AR software (Nikon) and mitochondrial morphology was classified as elongated, intermediate, or fragmented by at least two independent investigators, who were blinded to the sample information. Elongated mitochondria were those that have long tubulating or spreading reticular networks. Cells displaying mitochondria that are small spherical, ovoid, or short rod-shaped were presented as fragmented. Cells containing a mixture of small spherical and shorter tubular mitochondria were classified as intermediate.

Cell viability assay

Cells were seeded in 96-well cell culture plates (TPP Techno Plastic Products AG, Trasadingen, Switzerland) at a density of 4×10^3 cells per well. Cell viability was assessed using MTT assay (Thermo Fisher Scientific) according to the manufacturer's instructions. Cells were added with 0.4 mg/ml of MTT reagent and incubated for 4 h at 37 °C before the absorbance at 550 nm was measured with a microtiter plate reader (Bio-Rad).

Colony formation assay

1×10^3 cells were seeded in 6-well plates (Corning) and incubated with complete medium at 37 °C in 5% CO_2 for 24 h to facilitate their attachment. Subsequently, cells were treated with various concentrations of sodium arsenite for an additional 24 h period before the medium was removed and replaced with the fresh medium containing 10% FBS. After 14 days of incubation, cells were fixed in 4% PFA (Electron Microscopy Sciences) for 20 min and stained with 0.05% crystal violet (Sigma-Aldrich) for 2 h. Colonies were imaged and measured by the ImageJ software (NIH, Bethesda, MD, USA).

Flow cytometry

All experiments were performed using 6-well plates (Corning). A total of 10 000 events, excluding debris, were recorded for each sample. All flow cytometric data were obtained using BD Accuri C6 flow cytometer (BD Biosciences, San Jose, CA, USA) and analyzed by the FlowJo 7.6.1 software (FlowJo LLC, Ashland, OR, USA).

For measurement of mitochondrial membrane potential (ΔΨm) and mitochondrial reactive oxygen species (ROS), cells were treated with trypsin and prepared as single-cell suspensions. Cells were then stained either with 2 μM of the ΔΨm probe JC-1 (Invitrogen) for 20 min or with 5 μM of the mitochondrial superoxide indicator MitoSOX Red (Invitrogen) for 30 min following the

manufacturer's protocols. Cells were washed twice in ice-cold PBS before analysis with flow cytometry.

***TP53* gene statuses and clinical correlations**

Data on *TP53* gene statuses (WT and MUT *TP53*) used to analyze the associations between the presence of *TP53* mutations and the probabilities of metastases to lymph nodes (Fig. 1A) and distant organs (Fig. 1B) were derived from the TCGA Pan-Cancer and the Memorial Sloan-Kettering Integrated Mutation Profiling of Actionable Cancer Targets (MSK-IMPACT) [4] cohorts respectively, downloaded from the cBioPortal for Cancer Genomics (https://www.cbioportal.org/) [5, 6]. In each dataset, tumors were stratified into one of two categories: tumors with no *TP53* mutation (WT *TP53*), or tumors with one or more *TP53* mutations (MUT *TP53*). The complete TCGA Pan-Cancer dataset includes a total of 10 967 tumors from 10 953 patients across 32 different cancer types. *TP53* mutation data are available from 10 960 tumors. Among these tumor samples, 6 528 tumors have data on lymph node metastatic status (Fig. 1A) and 10 813 tumors have data on overall survival (Fig. 1C). The complete MSK-IMPACT cohort includes 10 945 tumors from 10 336 patients having both data on *TP53* mutations and tumor sites (Fig. 1B). The significance of the associations between the presence of *TP53* mutations and the probabilities of metastases to lymph nodes (Fig. 1A) and distant organs (Fig. 1B) was determined by Fisher's exact test. The significance of the difference in the Kaplan-Meier plot of the overall survival in patients with WT and MUT *TP53* (Fig. 1C) was determined by log-rank (Mantel-Cox) test.

Data on *TP53* mutation and DNA copy-number statuses used to analyze the associations between different subtypes of *TP53* alterations (*TP53* unaltered, missense mutation, and gene deletion) and lymph node metastases (Fig. S1) were derived from the TCGA Pan-Cancer, downloaded from the cBioPortal for Cancer Genomics (https://www.cbioportal.org/) [5, 6]. All tumors carrying missense mutations in the *TP53* gene were stratified into the *TP53* missense

group. Tumors with *TP53* DNA copy-number values less than -1 were stratified into the *TP53* gene deletion group. Tumors having WT *TP53* with DNA copy-number values less than 2 and greater than -1 were stratified into the *TP53* unaltered group. Copy-number values for the *TP53* gene were generated by the GISTIC algorithm and the GISTIC score was used to determine the copy-number (CN) status of *TP53*: CN = 0 was considered as diploid, CN < -1 was considered as copy-number loss (deletion), and CN > 2 was considered as copy-number gain (amplification). The significance of the associations between different subtypes of *TP53* alterations and lymph node metastases (Fig. S1) was determined by Fisher's exact test.

Differential protein expression analysis

Comparative analyzes of p53 protein expression levels (Fig. 1D, E) were performed using the reverse-phase protein array (RPPA) data acquired from the TCGA Pan-Cancer dataset in the cBioPortal for Cancer Genomics (https://www.cbioportal.org/) [5, 6]. A total of 4 741 tumors harboring WT *TP53* and having RPPA data were retrieved. Of which, 2 934 tumors have data on lymph node metastatic status (Fig. 1D) and 3 086 tumors have data on disease stages (Fig. 1E). RPPA values for p53 expression levels were stratified according to the lymph node metastatic status and the stage of the corresponding patient tumor. Mean protein expression levels of p53 were determined in lymph node-negative (N0) and -positive (N1+) (Fig. 1D) or earlier-stage (stage I+II) and advanced-stage (stage III+IV) (Fig. 1E) groups and significant downregulation of p53 protein expression in N1+ and advanced-stage tumors were statistically analyzed by two-tailed Student's t test (unpaired).

Differential gene expression analysis

Comparative analysis of gene expression patterns of p53 (Fig. 1F), Drp1 (Fig. 2E), and MMP9 (Fig. S5G) between primary and metastatic melanoma was performed using the RNA-Seq by Expectation-Maximization (RSEM) data extracted from TCGA in The UCSC Xena Browser

(http://xena.ucsc.edu/) [7]. Pan-Cancer analyzes of the differential gene expression of Drp1 (Fig. 2C, D) and MMP9 (Fig. 6H, I) among tumor samples obtained from patients with lymph node-negative (N0) and -positive (N1+) prognostics (Fig. 2C and 6H) or at different stages (stage I+II and III+IV) (Fig. 2D and 6I); and that of MMP9 (Fig. S5E) among tumor samples with WT and MUT *TP53* were performed using RSEM values acquired from the TCGA Pan-Cancer dataset in the cBioPortal for Cancer Genomics (https://www.cbioportal.org/) [5, 6]. RSEM data are available for 10 071 tumors. Of these tumors, 6 445 tumors have data on lymph node metastatic status (Fig. 2C and 6H), 6 613 tumors have data on cancer stages (Fig. 2D and 6I), and 10 070 tumors have data on *TP53* mutation status (Fig. S5E).

All RSEM values were log2 transformed. Similar to methods used for protein expression data, mean expression levels were determined for mRNAs in individual groups and significant up- or down-regulation of mRNA expression was statistically analyzed by two-tailed Student's t test (unpaired).

Correlation analysis

The correlation between protein expression levels of p53 versus mRNA expression levels of MMP9 (Fig. S5F) was determined using RPPA and RSEM values extracted from the TCGA Pan-Cancer dataset in the cBioPortal for Cancer Genomics (https://www.cbioportal.org/) [5, 6]. All RSEM values were log2 transformed. Of the 10 071 tumors with available RSEM data, 4 538 tumors harbor WT *TP53* and have RPPA data.

For the correlations between p53 protein expression levels versus 4E-BP1 S65, 4E-BP1 T37/T46, and mTOR S2448 phosphorylation levels (Fig. 5A); or ERK1/2 T202/Y204 phosphorylation levels versus p53 protein levels and phosphorylation levels of 4E-BP1 S65, 4E-BP1 T37/T46, and mTOR S2448 (Fig. 6A), we used level 4 normalized RPPA data of the TCGA Pan-Cancer cohort downloaded from The Cancer Proteome Atlas (https://tcpaportal.org/). The Pearson correlation

coefficient (r) was used to establish the correlations between expression levels of proteins versus mRNAs, proteins versus proteins, and mRNAs versus mRNAs and determine the *p-value*.

Survival analysis

For correlation analysis of p53 protein expression levels and the overall survival of cancer patients harboring WT *TP53* (Fig. 1G), publicly available RPPA data and patients' overall survival status from the TCGA Pan-Cancer dataset were downloaded from the cBioPortal for Cancer Genomics (https://www.cbioportal.org/) [5, 6]. Of tumors with WT *TP53*, 4 700 tumors have both RPPA and overall survival data. Patients were split into high and low p53 expression groups based on the median values of p53 protein expression. The significance of the difference in the overall survival of patients with high and low p53 protein expression in Kaplan-Meier plots was determined by log-rank (Mantel-Cox) test.

For correlation analysis of mRNA expression levels of Drp1 (Fig. 2F) and MMP9 (Fig. S5H) versus overall survival of cancer patients, RSEM values and patients' overall survival data from the TCGA Pan-Cancer dataset were downloaded from the cBioPortal for Cancer Genomics (https://www.cbioportal.org/) [5, 6]. A total of 9 994 tumors having both RSEM and overall survival data were retrieved. Similar to methods used for the analysis of correlation of p53 protein expression patterns and overall survival, patients were split into high and low expression groups based on the median values of Drp1 and MMP9 mRNA expression. The significance of the difference in the overall survival of patients with high and low expressions of individual mRNAs in Kaplan-Meier plots was determined by log-rank (Mantel-Cox) test.

Supplementary Information accompanies the paper on the *Oncogenesis* website (http://www.nature.com/oncsis).

References for Supplementary Information

1 Kuo M-H, Lee A-C, Hsiao S-H, Lin S-E, Chiu Y-F, Yang L-H *et al*. Cross-talk between SOX2 and TGFβ Signaling Regulates EGFR–TKI Tolerance and Lung Cancer Dissemination. *Cancer Research* 2020; 80: 4426-4438.

2 Liang C-C, Park AY, Guan J-L. In vitro scratch assay: a convenient and inexpensive method for analysis of cell migration in vitro. *Nature Protocols* 2007; 2: 329-333.

3 Liu Y, Zhao X, Ding J, Xing Y, Zhou M, Wang X *et al*. Evidence of Accumulated Endothelial Progenitor Cells in the Lungs of Rats with Pulmonary Arterial Hypertension by (89)Zr-oxine PET Imaging. *Mol Ther Methods Clin Dev* 2020; 17: 1108-1117.

4 Zehir A, Benayed R, Shah RH, Syed A, Middha S, Kim HR *et al*. Mutational landscape of metastatic cancer revealed from prospective clinical sequencing of 10,000 patients. *Nat Med* 2017; 23: 703-713.

5 Cerami E, Gao J, Dogrusoz U, Gross BE, Sumer SO, Aksoy BA *et al*. The cBio Cancer Genomics Portal: An Open Platform for Exploring Multidimensional Cancer Genomics Data. *Cancer Discovery* 2012; 2: 401-404.

6 Gao J, Aksoy BA, Dogrusoz U, Dresdner G, Gross B, Sumer SO *et al*. Integrative Analysis of Complex Cancer Genomics and Clinical Profiles Using the cBioPortal. *Science Signaling* 2013; 6: pl1-pl1.

7 Goldman MJ, Craft B, Hastie M, Repečka K, McDade F, Kamath A *et al*. Visualizing and interpreting cancer genomics data via the Xena platform. *Nature Biotechnology* 2020; 38: 675-678.

Supplementary Figure Legends

Fig. S1 *TP53* missense mutations exhibit gain-of-function effects on lymph node metastases. Contingency analysis of the associations between the presence of different *TP53* alterations (*TP53* unaltered, missense mutation, and gene deletion) and the probabilities of metastases to lymph nodes. N0, lymph node-negative; N1+, lymph-node-positive. Data were derived from The Cancer Genome Atlas (TCGA) Pan-Cancer cohort. The p values represent the significance of the observed mutual exclusivity between different subtypes of *TP53* alterations (*TP53* unaltered versus *TP53* missense mutation, *TP53* unaltered versus *TP53* deletion, and *TP53* missense mutation versus *TP53* deletion), as analyzed by Fisher's exact test.

Fig. S2 Effects of p53 on cell viability, mitochondrial integrity, and cell migration. (A) Immunoblot of p53 in A549 cells treated with SA at the indicated concentrations for 24 h. GAPDH was used as a loading control. (B and C) The viability of A549 cells treated with SA at the indicated concentrations for 24 h was measured by (B) colony formation assay or (C) MTT assay. (D) Cell viability was measured by MTT assay in siCtrl- and sip53-transfected A549 cells with and without 20 μM SA treatment for 24 h. (E and F) Flow cytometry analysis of (E) mitochondrial membrane potential ($\Delta\Psi m$) or (F) mitochondrial ROS levels in siCtrl, siCtrl+SA, sip53, and sip53+SA A549 cells. (G) Quantification (left) and representative images (right) of the area in a wound-healing assay covered by Ctrl and p53-expressing H1299 cells. Scale bar: 100 μm. Error bars represent mean ± SD. Data were analyzed by one-way ANOVA with Tukey's multiple comparisons test (B-F) or two-tailed unpaired Student's t test (G).

Fig. S3 p53 suppresses cell motility by inhibiting the pro-fission phosphorylation of Drp1. (A) qRT-PCR analysis of the mRNA levels of p53 and genes involved in mitochondrial fusion (Mfn1 (Mitofusin 1), Mfn2 (Mitofusin 2), and Opa1 (Optic Atrophy 1)) and fission (Drp1, Fis1 (mitochondrial fission 1), Mff (mitochondrial fission factor), and MIEF1 (mitochondrial elongation

factor 1)) in sip53 A549 cells. Results are expressed relative to those in siCtrl A549 cells (dashed line). (B) Immunoblot of the indicated proteins in Ctrl and p53 H1299 cells with and without 20 μM SA treatment for 24 h. GAPDH was used as a loading control. (C) Quantification of the levels of Drp1, p-Drp1 (S637), and p-Drp1 (S616) in Fig. S3B. (D and E) Migration distance (D) and representative trajectories (E) of siCtrl (n = 29), siCtrl+SA (n = 17), sip53 (n = 13), sip53+SA (n = 15), sip53+siDrp1 (n = 24), and sip53+siDrp1+SA (n = 24) A549 cells. (F) Quantification (left) and representative images (right) of the area in a wound-healing assay covered by siCtrl, siCtrl+SA, sip53, sip53+SA, sip53+siDrp1, and sip53+siDrp1+SA A549 cells. Scale bar: 100 μm. Error bars represent mean ± SD. Data were analyzed by two-tailed unpaired Student's t test (A and C) or one-way ANOVA with Tukey's multiple comparisons test (D and F). ****, $p < 0.0001$; ns, not significant.

Fig. S4 p53 transcriptional activity participates in regulating mTORC1-controlled MTFP1 protein levels affecting cell motility. (A) Immunoblot of the indicated proteins in control (Ctrl) and PFT-α-treated (PFT-α) A549 cells with and without 20 μM SA treatment for 24 h. GAPDH was used as a loading control. (B) qRT-PCR analysis of the mRNA levels of p53 and p53 downstream target genes in sip53 A549 cells. Results were expressed relative to those in siCtrl A549 cells (dashed line). (C) qRT-PCR analysis of mRNA expression of MTFP1 in siCtrl and sip53 A549 cells with and without 20 μM SA treatment for 24 h. (D and E) Migration distance (D) and representative trajectories (E) of siCtrl (n = 28), siCtrl+SA (n = 17), sip53 (n = 13), sip53+SA (n = 14), sip53+simTOR (n = 33), sip53+simTOR+SA (n = 28), sip53+siMTFP1 (n = 32), and sip53+siMTFP1+SA (n = 20) A549 cells. Error bars represent mean ± SD. Data were analyzed by two-tailed unpaired Student's t test (B) or one-way ANOVA with Tukey's multiple comparisons test (C and D). ***, $p < 0.001$; ****, $p < 0.0001$.

Fig. S5 p53 controls cell motility and MMP9 expression through mTOR/MTFP1/Drp1/ERK1/2 signaling axis. (A) Phase-contrast imaging of siCtrl, sip53, sip53+siDrp1, sip53+simTOR, sip53+siMTFP1, and sip53+PD98059 A549 and MCF-7 cells. Scale bar: 100 μm. (B and C) Migration distance (B) and representative trajectories (C) of siCtrl (n = 31), siCtrl+SA (n = 19), sip53 (n = 13), sip53+SA (n = 14), sip53+ PD98059 (n = 20), and sip53+PD98059+SA (n = 35) A549 cells. (D) qRT-PCR analysis of MMP9 mRNA expression in siCtrl, sip53, sip53+PD98059, sip53+siDrp1, sip53+siMTFP1, and sip53+simTOR MCF-7 cells. (E) MMP9 mRNA expression in tumors having WT and MUT *TP53*. (F) Correlation between p53 protein levels and MMP9 mRNA levels in tumors having WT *TP53* (n = 4 538 samples). (G) MMP9 mRNA levels in primary and distant metastatic melanoma. (H) Kaplan-Meier analysis of the overall survival in cancer patients with low and high MMP9 mRNA expression levels (left). Patients were further divided into MMP9 mRNA high / WT p53 protein low and MMP9 mRNA low / WT p53 protein high groups (right). Data were extracted from TCGA (E-H). Error bars represent mean ± SD (D) or SEM (E and G). Data were analyzed by one-way ANOVA with Tukey's multiple comparisons test (B and D), two-tailed unpaired Student's t test (E and G), or log-rank test (H).

Supplementary Figures

Figure S1

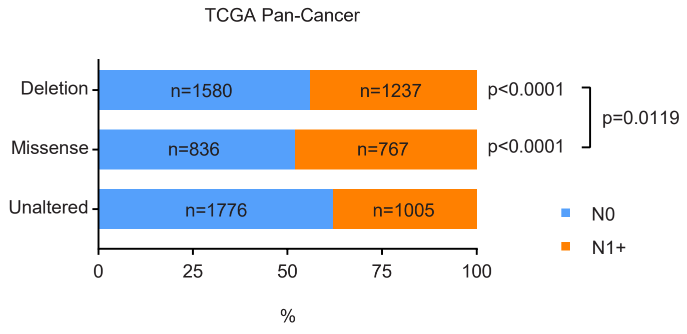

Figure S2

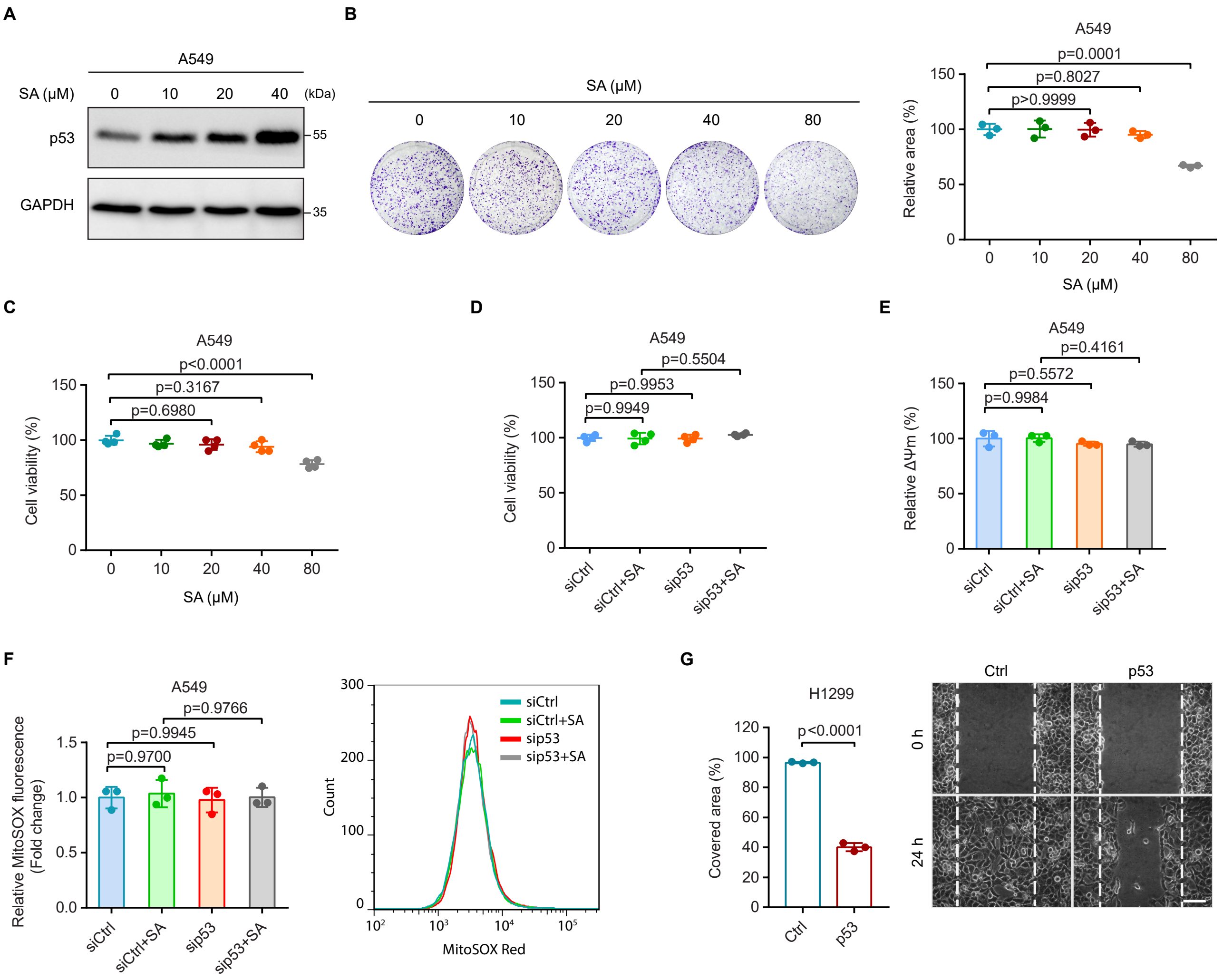
A
A549
SA (μM)
0
10
20
40
(kDa)
p53
55
GAPDH
35
B
SA (μM)
0
10
20
40
80
A549
p=0.0001
p=0.8027
p>0.9999
Relative area (%)
SA (μM)
C
A549
p<0.0001
p=0.3167
p=0.6980
Cell viability (%)
SA (μM)
D
A549
p=0.5504
p=0.9953
p=0.9949
Cell viability (%)
siCtrl
siCtrl+SA
sip53
sip53+SA
E
A549
p=0.4161
p=0.5572
p=0.9984
Relative ΔΨm (%)
F
A549
p=0.9766
p=0.9945
p=0.9700
Relative MitoSOX fluorescence
(Fold change)
Count
MitoSOX Red
G
H1299
p<0.0001
Covered area (%)
Ctrl
p53
0 h
24 h

Figure S3

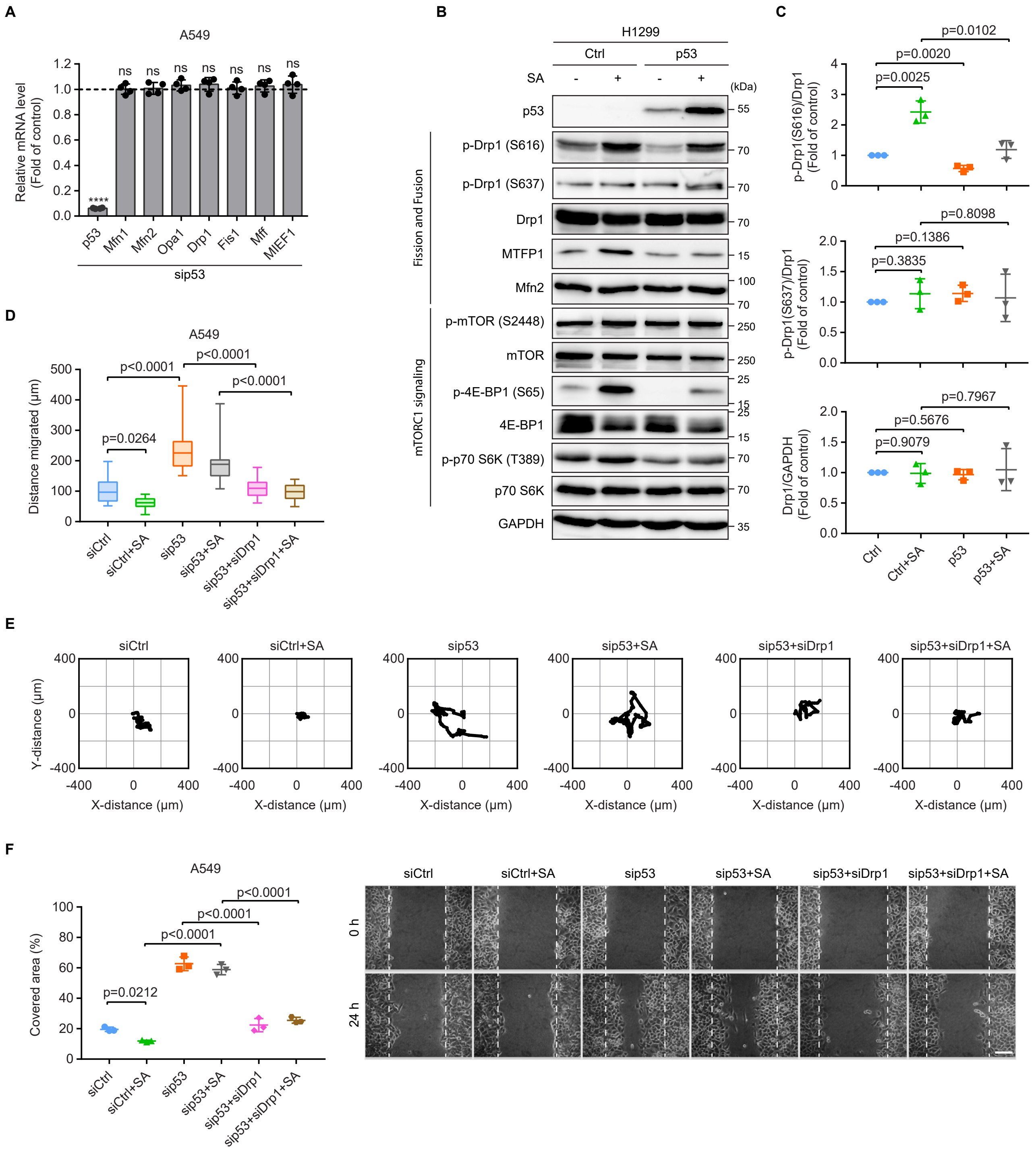
A
A549
Relative mRNA level (Fold of control)
****
ns
p53
Mfn1
Mfn2
Opa1
Drp1
Fis1
Mff
MIEF1
sip53
B
H1299
Ctrl
p53
SA
(kDa)
p53
p-Drp1 (S616)
p-Drp1 (S637)
Drp1
MTFP1
Mfn2
p-mTOR (S2448)
mTOR
p-4E-BP1 (S65)
4E-BP1
p-p70 S6K (T389)
p70 S6K
GAPDH
Fission and Fusion
mTORC1 signaling
C
p-Drp1(S616)/Drp1 (Fold of control)
p=0.0102
p=0.0020
p=0.0025
p-Drp1(S637)/Drp1 (Fold of control)
p=0.8098
p=0.1386
p=0.3835
Drp1/GAPDH (Fold of control)
p=0.7967
p=0.5676
p=0.9079
Ctrl
Ctrl+SA
p53
p53+SA
D
A549
Distance migrated (μm)
p<0.0001
p<0.0001
p<0.0001
p=0.0264
siCtrl
siCtrl+SA
sip53
sip53+SA
sip53+siDrp1
sip53+siDrp1+SA
E
siCtrl
siCtrl+SA
sip53
sip53+SA
sip53+siDrp1
sip53+siDrp1+SA
Y-distance (μm)
X-distance (μm)
F
A549
Covered area (%)
p<0.0001
p<0.0001
p<0.0001
p=0.0212
siCtrl
siCtrl+SA
sip53
sip53+SA
sip53+siDrp1
sip53+siDrp1+SA
0 h
24 h

Figure S4

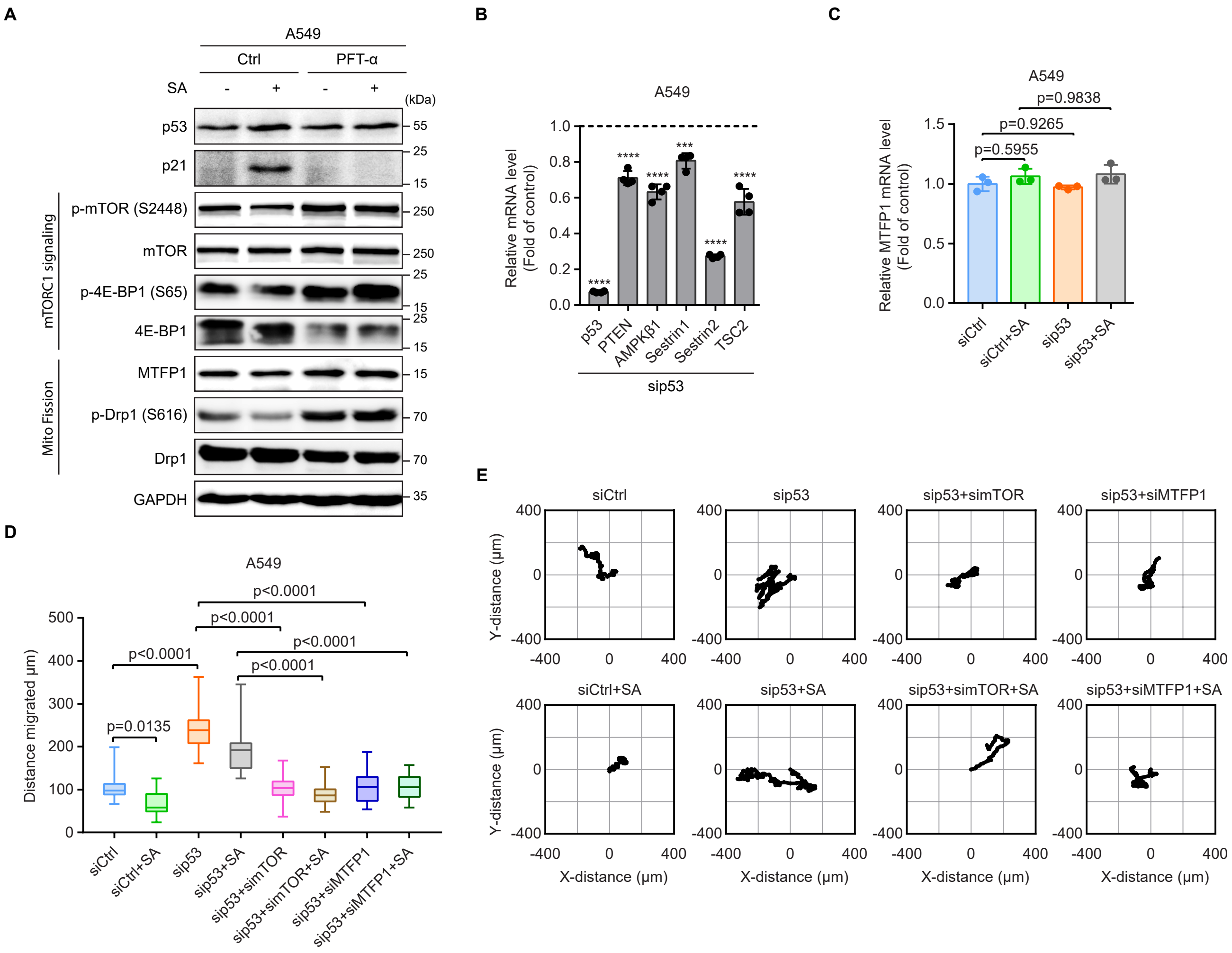
A
A549
Ctrl
PFT-α
SA
(kDa)
p53
p21
p-mTOR (S2448)
mTOR
p-4E-BP1 (S65)
4E-BP1
MTFP1
p-Drp1 (S616)
Drp1
GAPDH
mTORC1 signaling
Mito Fission
B
A549
Relative mRNA level (Fold of control)
p53
PTEN
AMPKβ1
Sestrin1
Sestrin2
TSC2
sip53
C
A549
Relative MTFP1 mRNA level (Fold of control)
p=0.9838
p=0.9265
p=0.5955
siCtrl
siCtrl+SA
sip53
sip53+SA
D
A549
Distance migrated (μm)
p<0.0001
p=0.0135
siCtrl
siCtrl+SA
sip53
sip53+SA
sip53+simTOR
sip53+simTOR+SA
sip53+siMTFP1
sip53+siMTFP1+SA
E
siCtrl
sip53
sip53+simTOR
sip53+siMTFP1
siCtrl+SA
sip53+SA
sip53+simTOR+SA
sip53+siMTFP1+SA
Y-distance (μm)
X-distance (μm)

Figure S5

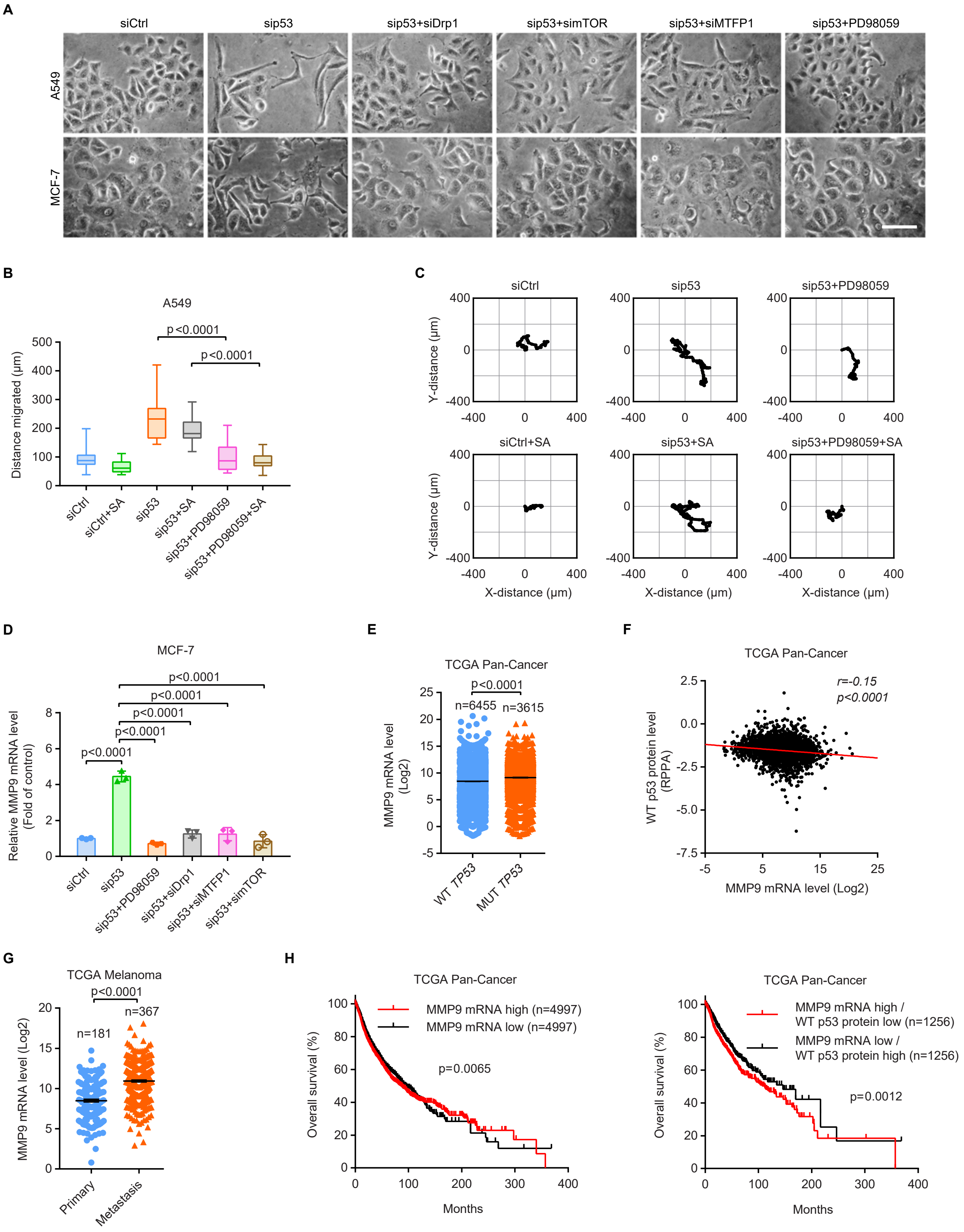
A
siCtrl
sip53
sip53+siDrp1
sip53+simTOR
sip53+siMTFP1
sip53+PD98059
A549
MCF-7
B
A549
Distance migrated (μm)
p<0.0001
p<0.0001
siCtrl
siCtrl+SA
sip53
sip53+SA
sip53+PD98059
sip53+PD98059+SA
C
siCtrl
sip53
sip53+PD98059
siCtrl+SA
sip53+SA
sip53+PD98059+SA
Y-distance (μm)
X-distance (μm)
D
MCF-7
Relative MMP9 mRNA level
(Fold of control)
p<0.0001
p<0.0001
p<0.0001
p<0.0001
p<0.0001
siCtrl
sip53
sip53+PD98059
sip53+siDrp1
sip53+siMTFP1
sip53+simTOR
E
TCGA Pan-Cancer
MMP9 mRNA level
(Log2)
p<0.0001
n=6455
n=3615
WT TP53
MUT TP53
F
TCGA Pan-Cancer
WT p53 protein level
(RPPA)
r=-0.15
p<0.0001
MMP9 mRNA level (Log2)
G
TCGA Melanoma
MMP9 mRNA level (Log2)
p<0.0001
n=181
n=367
Primary
Metastasis
H
TCGA Pan-Cancer
Overall survival (%)
MMP9 mRNA high (n=4997)
MMP9 mRNA low (n=4997)
p=0.0065
Months
TCGA Pan-Cancer
MMP9 mRNA high /
WT p53 protein low (n=1256)
MMP9 mRNA low /
WT p53 protein high (n=1256)
p=0.0012
Months

Supplementary Tables

Table S1. siRNA sequences

**Target protein**	**siRNA ID**	**Target sequence (5'-3')**
p53	VHS40367	Sense: CCAGUGGUAAUCUACUGGGACGGAA
		Antisense: UUCCGUCCCAGUAGAUUACCACUGG
p53	VHS40366	Sense: CCAUCCACUACAACUACAUGUGUAA
		Antisense: UUACACAUGUAGUUGUAGUGGAUGG
Drp1	HSS115288	Sense: CCUGCUUUAUUUGUGCCUGAGGUUU
		Antisense: AAACCUCAGGCACAAAUAAAGCAGG
mTOR	HSS103825	Sense: AGGACGCUCACAUUGCUAGAUGUGG
		Antisense: CCACAUCUAGCAAUGUGAGCGUCCU
MTFP1	HSS182106	Sense: GGGAUACCUGGGCUAUGCCAAUGAG
		Antisense: CUCAUUGGCAUAGCCCAGGUAUCGC
MMP9	L-005970-00-0005	ON-TARGETplus SMARTpool siRNA J-005970-07: GCAUAAGGACGACGUGAAU
		ON-TARGETplus SMARTpool siRNA J-005970-08: GGACCAAGGAUACAGUUUG
		ON-TARGETplus SMARTpool siRNA J-005970-09: GCGCUCAUGUACCCUAUGU
		ON-TARGETplus SMARTpool siRNA J-005970-10: GAACCAAUCUCACCGACAG

Table S2. List of primers used for qRT-PCR

Target protein	Sequence (5'-3')
p53	Forward: AAGGAAATTTGCGTGTGGAGT
	Reverse: AAAGCTGTTCCGTCCCAGTA
Mfn1	Forward: GAGGTGCTATCTCGGAGACAC
	Reverse: GCCAATCCCACTAGGGAGAAC
Mfn2	Forward: CACATGGAGCGTTGTACCAG
	Reverse: TTGAGCACCTCCTTAGCAGAC
Opa1	Forward: TGTGAGGTCTGCCAGTCTTTA
	Reverse: TGTCCTTAATTGGGGTCGTTG
Drp1	Forward: ACCCGGAGACCTCTCATTCT
	Reverse: TGACAACGTTGGGTGAAAAA
Fis1	Forward: GATGACATCCGTAAAGGCATCG
	Reverse: AGAAGACGTAATCCCGCTGTT
Mff	Forward: CACCACCTCGTGTACTTACGC
	Reverse: GTCTGCCAACTGCTCGGATTT
MIEF1	Forward: CACGGCCATTGACTTTGTGC
	Reverse: TCGTACATCCGCTTAACTGCC
PTEN	Forward: AGTTCCCTCAGCCGTTACCT
	Reverse: AGGTTTCCTCTGGTCCTGGT
AMPKβ1	Forward: TCCGATGTGTCTGAGCTGTC
	Reverse: GTTCAGCATGACGTGATTGG
Sestrin1	Forward: AGCCCATAGACCTTGGCTTA
	Reverse: TCCACACTGTGATTGCCATT

Sestrin2	Forward: TGCTGTGCTTTGTGGAAGAC
	Reverse: GCTGCCTGGAACTTCTCATC
TSC2	Forward: TGCAAGCCGTCTTCCACAT
	Reverse: ATGGACACAAAGTCGTTGC
MTFP1	Forward: CCATCCCCATCATTATCCAC
	Reverse: TTCCCCACTGTTGGGTAGAG
Snail	Forward: GCGAGCTGCAGGACTCTAAT
	Reverse: CCACTGTCCTCATCTGACA
Slug	Forward: GGGGAGAAGCCTTTTTCTTG
	Reverse: TCCTCATGTTTGTGCAGGAG
ZEB2	Forward: TTCCTGGGCTACGACCATAC
	Reverse: TGTGCTCCATCAAGCAATTC
FOXC2	Forward: GCCTAAGGACCTGGTGAAGC
	Reverse: TTGACGAAGCACTCGTTGAG
EPCAM	Forward: GCAGCTCAGGAAGAATGTG
	Reverse: CAGCCAGCTTTGAGCAAATGAC
MMP9	Forward: GGGACGCAGACATCGTCATC
	Reverse: TCGTCATCGTCGAAATGGGC
GAPDH	Forward: TGCACCACCAACTGCTTAGC
	Reverse: GGCATGGACTGTGGTCATGAG
